# Differences in the Relationship Between Educational Attainment and Health Status Across Racial and Ethnic Groups in a Multi-ethnic United States Older Adult Population: A Cross-Sectional Electronic Health Record-Based Study

**DOI:** 10.7759/cureus.73288

**Published:** 2024-11-08

**Authors:** Nancy P Gordon, Michelle Pimentel

**Affiliations:** 1 Division of Research, Kaiser Permanente, Pleasanton, USA; 2 Operations, Rock Medicine, Orange, USA

**Keywords:** copd, coronary artery disease, diabetes, educational attainment, educational attainment health disparities, hypertension, older adults, racial/ethnic health disparities, smoking

## Abstract

Introduction

We aimed to describe the relationship of educational attainment with the prevalence of six health outcomes (ever and current smoking, diabetes, hypertension, coronary artery disease, and chronic obstructive pulmonary disease) in an older adult population, including whether education-health relationships differed by health outcome, by racial and ethnic (racial/ethnic) group, and by racial/ethnic group within the same level of education.

Methods

This cross-sectional study used 2015-2016 electronic health record data for 149,417 non-Hispanic White (White), 15,398 African-American or other Black (Black), 15,319 Hispanic or Latino (Latino), 10,133 Filipino, and 8810 Chinese Northern California health plan members aged 65-79 years whose preferred language was English. For each racial/ethnic group, sex-specific age-standardized prevalence of the six health outcomes was estimated for four levels of education (non-high school graduate, high school graduate, some college, college graduate). Age-adjusted prevalence ratios were used to compare the prevalence between adjacent levels of education and at lower versus college graduate levels within racial/ethnic groups, and the prevalence between White adults and adults in the other racial/ethnic groups, within each level of education and overall.

Results

The education-health relationship varied across racial/ethnic groups and health outcomes, with gradient relationships more consistently seen for White, Black, and Latino older adults than Filipino and Chinese older adults. Even when a gradient relationship was not observed, the prevalence at the college graduate level was usually significantly lower than the prevalence at the three lower levels of education. The prevalence of current smoking, diabetes, and hypertension was higher among Black than White adults at most levels of education. Controlling for education level minimally affected comparisons of overall prevalence of health outcomes between adults in the White and the other racial/ethnic groups, with the broadest impact seen for Latino-White comparisons.

Conclusions

The relationship of level of education and health outcomes differs across racial/ethnic groups and by health outcome. This should be taken into consideration when using education as a risk adjustment factor or predictor of health outcomes in multi-ethnic older adult populations.

## Introduction

Educational attainment is an important social determinant of health and well-being in US adults [1]. Higher educational attainment is associated with greater health literacy, more health-promoting attitudes, greater future orientation, and healthier behaviors and lifestyles [1-5]. Adults with high educational attainment are also more likely than adults with less education to have grown up in, lived in, and worked in social and physical environments conducive to good health and to have greater financial resources to live a healthy lifestyle and afford good health care [1,2,5]. These personal, economic, and environmental factors jointly lead to a lower risk of developing chronic health conditions, better management of chronic conditions, and longer life expectancy [1-4,6-8].

Most research about the association of education with health, with the exception of mortality studies, has been based on self-reported data about health and healthcare use collected through surveys and clinical studies. While studies based on self-reported health data have provided valuable insights into education as a social determinant of health, there are several factors that may limit the validity and generalizability of these study results to contemporary populations, including study participation bias (i.e., lower income, less educated, and minority adults are less likely to participate in surveys and clinical studies and thus less likely to be described by the results); inaccuracy of self-reported health data; geographic variation in health-related behaviors/lifestyle factors and chronic disease prevalence that may confound the relationship of education and health; use of decades-old data that may not accurately reflect cultural changes such as population-level increases in educational attainment, dramatic decreases in the prevalence of smoking, and expanded healthcare access that have led to reductions in the prevalence of chronic health conditions such as diabetes and coronary artery disease (CAD).

In 2014, the US National Academy of Medicine (formerly Institute of Medicine) Subcommittee on Social and Behavioral Determinants of Health recommended that educational attainment be captured in the electronic health record (EHR) for all adults [9]. Following this, in 2016, a task force of the US National Academies of Sciences, Engineering, and Medicine (NASEM) recommended that educational attainment be considered for use as a risk adjustor for value-based Medicare payment programs [10]. However, survey-based studies examining the education-health relationship have generally been based on data from study populations comprised of broad age ranges (young, middle-aged, and older) and predominantly non-Hispanic White (White) adults. The results of studies that model the association of educational attainment with smoking and chronic conditions after controlling for age, sex, and race/ethnicity may not be generalizable to older adults and in particular, adults in racial/ethnic groups other than White. Cutler and Lleras-Muney [2] found that the effect of education on health and mortality in the US adult population seemed to diminish starting in late middle age, perhaps due to selective survival of the better-educated and nearly universal healthcare coverage through Medicare. There is also some evidence that the relationship between level of education with health behaviors (e.g., smoking) and development of chronic health conditions (e.g., diabetes and cardiovascular disease) may differ across racial and ethnic (racial/ethnic) groups. Some studies have shown significant racial/ethnic group differences in health behaviors and health outcomes among adults with the same level of education [7,11-14] and in the gap in the prevalence of health behaviors and health outcomes between college graduates and non-high school graduates [14]. Some researchers have also theorized that due to structural racism, segregation, and social stratification, there is a lesser protective association of higher educational attainment with good health for Black and Latino adults than White adults, a pattern termed minorities’ diminished returns (MDR) [15-17].

The goal of this study was to elucidate the relationship between educational attainment and the prevalence of smoking and chronic health conditions in a racial/ethnically diverse older adult population. Our research had three main aims. First, we wanted to learn whether, in this diverse older adult population, the pattern of the association of level of education with the prevalence of smoking and chronic conditions would differ across racial/ethnic groups. Second, we wanted to learn the extent to which the prevalence of these health outcomes would vary across racial/ethnic groups and by sex among adults within the same level of education. Third, we wanted to learn whether educational attainment would be a consistently impactful risk adjustor when comparing the prevalence of health outcomes across racial/ethnic groups. This EHR-based study focused on White, African-American or other Black (Black), Hispanic or Latino (Latino), Filipino, and Chinese adults aged 65-79 years, the five largest adult racial/ethnic groups in our Northern California health plan membership. The study examined the education-health relationship for six health outcomes that were previously shown to be associated with educational attainment, including ever and current smoking [18-20], diabetes [21-23], hypertension [24], CAD [25,26], and chronic obstructive pulmonary disease (COPD) [27].

## Materials and methods

Setting

Kaiser Permanente in Northern California (KPNC) is a vertically integrated healthcare delivery system that serves over 3 million adult members, including 700,000 adults aged ≥ 65, at medical facilities in the San Francisco Bay Area, Silicon Valley, Sacramento area, and Central Valley in California. The sociodemographically diverse KPNC adult membership is very similar to the insured adult population of Northern California with regard to demographic and health characteristics [28].

Study population

**Figure 1:**
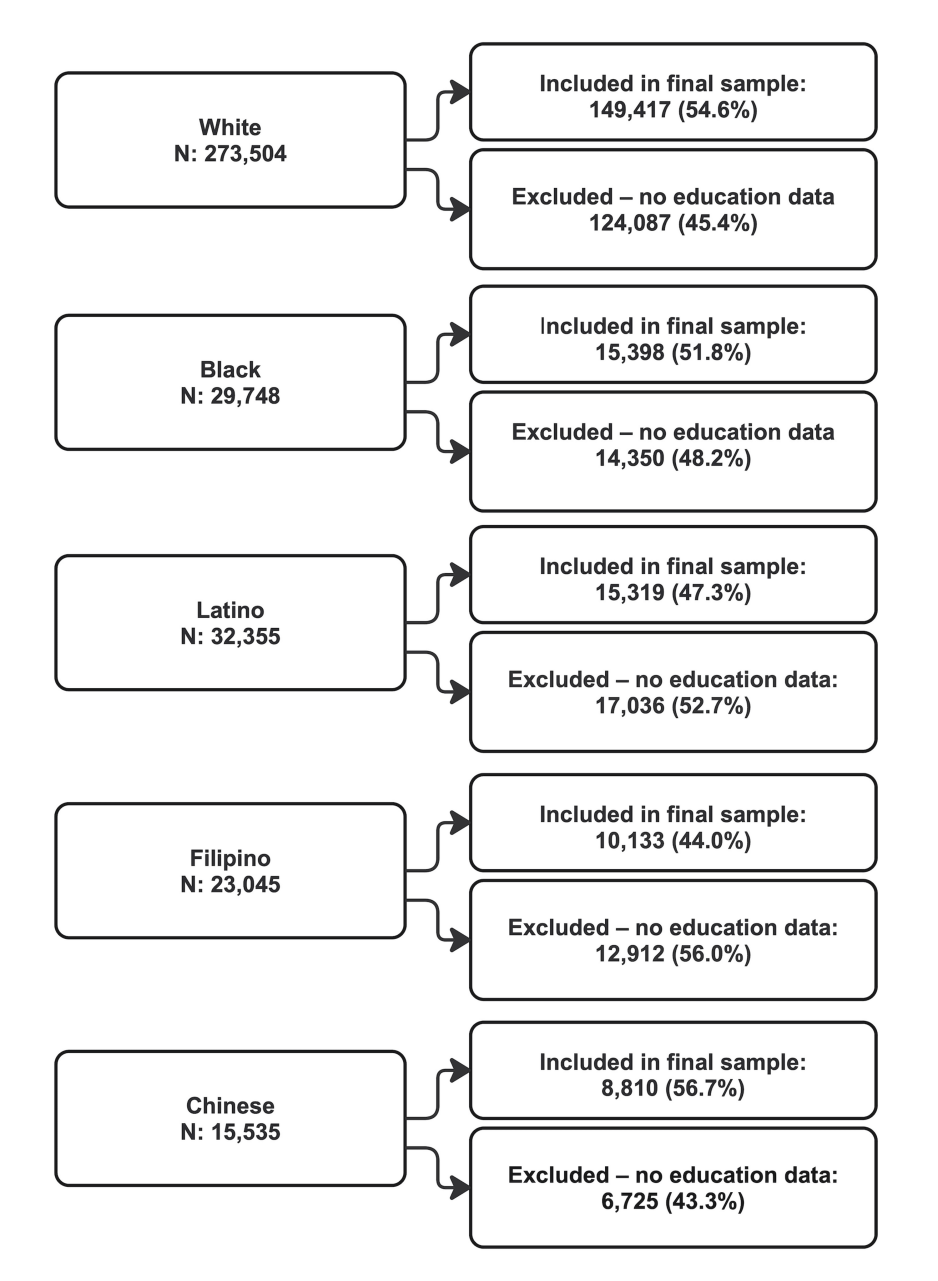
Study population flow diagram showing sample exclusions.

This study used a subset of the EHR-derived DECKA2016 research cohort created to study racial/ethnic differences in health and healthcare utilization in an adult health plan population [29]. The study sample included 149,417 White, 15,398 Black, 15,319 Latino, 10,133 Filipino, and 8810 Chinese adults who were aged 65-79 years in December 2016, had English as their preferred spoken language and could be assigned to one of four levels of education. Over half (53.1%) of adults in these racial/ethnic groups who met the English language criterion had education data, but this percentage differed by racial/ethnic group (54.5% of White, 51.8% of Black, 47.3% of Latino, 44.0% of Filipino, and 56.7% of Chinese adults) (Figure 1). Our final sample excluded adults with a non-English language preference due to sparse education data, other racial/ethnic groups due to insufficient sample size for our planned analyses, and adults aged >79 years to keep the age distribution relatively similar across racial/ethnic groups.

Study variables

*Demographic Variables*

Level of education was derived from data obtained from research and operations surveys, an onboarding questionnaire completed by some older adults at the time of joining KPNC’s Medicare Advantage program, and free text data found in the education field of the EHR. We categorized people into four levels of education: non-high school graduate, high school graduate/General Educational Development (GED) or post-high school technical/trade school (high school graduate), some college but no degree or an associate degree (some college), or bachelor’s degree or higher (college graduate), prioritizing self-reported education data from surveys and questionnaires over EHR text entries. Race and ethnicity had been assigned to all cohort members for the earlier study [29]. Age reflects age on December 31, 2016, based on date of birth, and sex reflects birth sex.

Health Outcomes

Smoking history was examined for current smoking and ever smoking based on EHR tobacco use data recorded at a visit closest to December 1, 2016. Ever smokers were adults who either currently smoked or had quit smoking in the past. When adults had no smoking status data or only “non-smoker” status entered during 2015-2016, we extended data abstraction to March 2012-March 2017. Smoking history was captured for over 99% of all racial/ethnic groups. Diabetes status was assigned based on inclusion in the KPNC Diabetes Registry by the end of 2016, as identified through inpatient and outpatient diagnoses, laboratory criteria, and medication use [30]. Hypertension, coronary artery disease, and COPD status were assigned based on qualifying International Classification of Diseases (ICD)-9 or ICD-10 codes for the condition during 2015 or 2016 from an outpatient visit or from the patient’s 2016 active problem list. See Table 1 for a listing of diagnosis codes used to assign chronic condition status.

**Table 1:** Diagnosis codes used to assign chronic conditions. ^a^ Karter et al. [30]. KPNC: Kaiser Permanente in Northern California; ICD: International Classification of Diseases; COPD: chronic obstructive pulmonary disease.

Condition	ICD-9 codes	ICD-10 codes
Diabetes mellitus	Entered into the KPNC Diabetes Registryby December 31, 2016 (based on inpatient or outpatient ICD-9/10 codes, lab test results, and medication use) ^a^	
Hypertension	362.11, 401.0, 401.1, 401.9, 402.00, 402.01, 402.10, 402.11, 402.90,402.91, 403.00, 403.01, 403.10, 403.11, 403.90, 403.91, 404.01, 404.03, 404.11, 404.13, 404.91, 404.93, 405.01, 405.09, 405.11, 405.19, 405.91, 405.99	I13.0, I13.1, I13.2, I13.9, I15.0, I15.8, I15.9, I10, I11.0, I11.9, I12.0, I12.9, H35.039
Coronary artery disease	410.00, 410.01, 410.02, 410.10, 410.11, 410.12, 410.20, 410.21, 410.22, 410.30, 410.31, 410.32, 410.40, 410.41, 410.42, 410.50, 410.51, 410.52, 410.60, 410.61, 410.62, 410.70, 410.71, 410.72, 410.80, 410.81, 410.82, 410.90, 410.91, 410.92, 411.0, 411.1, 411.81, 413.0, 413.1, 413.9, 414.02, 414.03, 414.04, 414.05, 414.06, 414.07, 414.2, 414.8, 414.9	I20.0, I20.1, I20.8, I20.9, I21.02, I21.09, I21.11, I21.19, I21.21, I21.29, I21.3, I21.4, I24.0, I24.1, I25.19, I25.82, I25.810, I25.811, I25.812, I25.5, I25.89, I25.9
COPD/chronic bronchitis	491.20, 491.21, 491.22, 492.0, 492.8, 493.20, 493.21, 493.22, 496, 518.1, 518.2	J44.0, J44.1, J44.9, J43.0, J43.1, J43.2, J43.8, J43.9, J98.2, J98.3

Statistical analysis

All analyses for this cross-sectional study were performed using SAS version 9.4 (SAS Institute, Cary, NC). To facilitate a direct comparison of racial/ethnic groups within the level of education unaffected by differences in the age composition of education-level subgroups, we standardized prevalence estimates within each level of education for all racial/ethnic groups and both sexes to the age distribution of the full study sample using three age groups (65-69 years: 0.3948; 70-74 years: 0.3484; 75-79 years: 0.2568). To assess whether differences in the prevalence between levels of education were statistically significant within and across racial/ethnic groups after adjusting for the same age groups used for age-standardization, we used modified log-Poisson regression models to produce adjusted prevalence ratios (aPR). The aPR compares the prevalence of a health outcome in one group to that in a reference group after adjusting for age using the same three age groups used for the age standardization. An aPR > 1.00 indicates that the comparison group has a higher prevalence than the reference group, an aPR < 1.00 indicates a lower prevalence, and the two groups are statistically significantly different at p <0.05 if the 95% confidence interval (CI) does not include 1.00. An aPR of 1.20 is interpreted as the comparison group having a prevalence 20% higher than the reference group, an aPR of 2.00 as a two-fold higher prevalence, and an aPR of 0.50 as a 50% lower prevalence. We made an *a priori *decision that a difference in prevalence between two subgroups (e.g., two levels of education, men, and women, or two racial/ethnic groups) would only be considered meaningfully significant if the aPR was statistically significant and the two age-standardized percentages differed by ≥ 1 percentage point. When interpreting the aPR, it is important to take into consideration the prevalence values being compared because differences in the prevalence of 1% versus 2% and 20% versus 40% would both have an aPR of 2.00 even though the gap in the prevalence is much wider in the latter comparison.

We first compared the prevalence of health outcomes among adults at the three lower levels of education to the prevalence among college graduates. Next, we examined the pattern of association of educational attainment with health outcomes by comparing the prevalence at each level of education to the prevalence at the next higher level of education (e.g., non-high school graduate versus high school graduate and high school graduate versus some college). When the prevalence at each consecutive level of education was consistently lower than the prevalence at the next higher level, educational attainment was considered to have a step-gradient relationship with the health outcome. When the prevalence at two adjacent levels of education did not meaningfully differ but an overall pattern of decreased prevalence with increasing education was observed, educational attainment was considered to have a non-step gradient relationship. We used the same meaningful significance criteria to evaluate differences in prevalence between men and women within the same racial/ethnic group and level of education, and racial/ethnic differences in prevalence within the same level of education (Black, Latino, Chinese, and Filipino adults versus White adults and Filipino adults versus Chinese adults). Models used to produce age-standardized prevalence estimates and aPRs for the full sample (not individual racial/ethnic groups) controlled for racial/ethnic groups in addition to age groups. Results reported in the text for “adults” indicate that the finding applies to both men and women.

## Results

Characteristics of the study population

Table 2 shows that Black, Latino, Filipino, and Chinese adults were more likely than White adults to be non-high school graduates. Black and Latino adults were less likely and Filipina women and Chinese adults were more likely to be college graduates. Among White, Black, Latino, and Chinese adults, men were more likely than women to be college graduates, but among Filipino adults, this was reversed. Table 2 also shows that there are significant differences between White adults and adults in the other racial/ethnic groups in smoking status and between men and women within racial/ethnic groups in the overall prevalence of most of the health outcomes studied. Compared to White adults, Black adults were more likely to be current and ever smokers and to have diabetes, hypertension, and COPD, with CAD prevalence higher among Black women but the same for Black men. Latina and Filipina women and Chinese adults were less likely to be current and ever smokers than White adults, with the prevalence among Latino and Filipino men similar to White men. The prevalence of diabetes was higher among Latino, Filipino, and Chinese adults than among White adults, with Filipino adults having the highest prevalence across all racial/ethnic groups. The prevalence of hypertension was higher among Latino and much higher among Filipino adults compared to White adults, but similar among Chinese adults. There was less racial/ethnic variation in the prevalence of CAD, but consistent with their lower prevalence of ever smoking, Latino and Chinese adults and Filipina women were less likely than their White counterparts to have COPD. Table 2 also shows that compared to Chinese adults, Filipina women were more likely and Filipino men were less likely to be college graduates. Filipino adults were also more likely than Chinese adults to be ever smokers and have diabetes, hypertension, CAD, and COPD, and Filipino men were more likely to be current smokers. Where sex differences in health outcomes were seen, the prevalence was always higher among men than women.

**Table 2:** Characteristics of the study population. n: number of adults; %: percentage of adults; GED: high school diploma equivalent; COPD: chronic obstructive pulmonary disease. ^a^ Percentages are based on data standardized to the distribution of ages 65-69, 70-74, and 75-79 years in the full sample. ^b^ Adults in this racial/ethnic group significantly differ from White adults based on p < 0.05 and a difference of ≥ 1 percentage point. ^c^ Filipino adults significantly differ from Chinese adults based on p < 0.05 and a difference of ≥ 1 percentage point. ^d^ Men significantly differ from women in the same racial/ethnic group based on p < 0.05 and a difference of ≥ 1 percentage point.

Characteristic	White		Black		Latino		Filipino		Chinese	
Women	n = 82,585		n = 9128		n = 8207		n = 5758		n = 4432	
	n	%	n	%	n	%	n	%	n	%
Age (prior to standardization)										
65-69 years	32146	38.9	3919	42.9^b^	3368	41.0^ b^	2263	39.3	2318	52.3^b^
70-74 years	29423	35.6	2928	32.1	2608	31.8	1963	34.1	1196	27.0
75-79 years	21016	25.5	2281	25.0	2231	27.2^ b^	1532	26.6^d^	918	20.7^b^
Level of education ^a^										
Non-high school graduate	2469	3.0	537	6.0^b^	1408	17.1^ b^	474	8.2^bc^	260	6.0^b^
High school graduate/GED	13334	16.2	1399	15.4	2170	26.5	546	9.5	500	11.9
Some college (no degree) or associate degree (AD)	28240	34.2	4268	46.8	2721	33.2	1284	22.3	1120	25.4
College graduate (≥ bachelor’s degree)	38542	46.7	2924	31.8^b^	1908	23.2^b^	3454	60.0^bc^	2552	56.7^b^
Prevalence of smoking and chronic health conditions ^a^										
Ever smoker	35484	43.1	4038	44.6^b^	2669	32.6^b^	785	13.7^bc^	526	12.4^b^
Current smoker	4200	5.1	739	8.1^b^	345	4.2	135	2.4^b^	70	1.5^b^
Diabetes	12533	15.2	3088	34.0^b^	2630	32.1^b^	2451	42.6^bc^	901	20.8^b^
Hypertension	44605	54.0	7320	80.5^b^	5303	64.7^b^	4560	79.1^bc^	2338	54.8
Coronary artery disease	2615	3.2	520	5.8^b^	354	4.3^b^	255	4.4^bc^	94	2.3
COPD	7021	8.5	862	9.6^b^	423	5.1^b^	227	3.9^bc^	99	2.4^b^
Men	n = 66832		n = 6270		n = 7112		n = 4375		n = 4378	
	n	%	n	%	n	%	n	%	n	%
Age (prior to standardization)										
65-69 years	25230	37.8	2614	41.7^b^	2798	39.3	1710	39.1	2194	50.1^b^
70-74 years	24204	36.2	2020	32.2	2350	33.1	1522	34.8	1154	26.4
75-79 years	17398	26.0	1636	26.1	1964	27.6^b^	1143	26.1	1030	23.5^bd^
Level of education ^a^										
Non-high school graduate	1934	2.9	405	6.5^b^	1139	15.9^bd^	240	5.5^bd^	205	4.8^bd^
High school graduate/GED	7790	11.6	1007	16.1	1496	21.0	523	11.9	355	8.1
Some college (no degree) or associate degree (AD)	19852	29.7	2681	42.7	2382	33.6	1248	28.6	966	22.1
College graduate (≥ bachelor’s degree)	37256	55.8^d^	2177	34.7^bd^	2095	29.5^bd^	2364	54.0^ c^^d^	2852	65.0^bd^
Prevalence of smoking and health conditions ^a^										
Ever smoker	34442	51.7^d^	3570	57.6^bd^	3695	52.2^d^	2514	57.8^bcd^	1253	29.6^bd^
Current smoker	3621	5.5	584	9.4^bd^	389	5.5^d^	254	5.8^ c^^d^	133	3.0^bd^
Diabetes	14752	22.0^d^	2374	37.9^bd^	2762	38.8^bd^	2210	50.5^bcd^	1205	28.2^bd^
Hypertension	40272	60.1^d^	4875	77.9^bd^	4866	68.2^bd^	3467	79.2^bc^	2603	60.8^d^
Coronary artery disease	5231	7.6^d^	499	7.3^d^	607	7.8^d^	429	8.9^bcd^	263	5.5^bd^
COPD	5895	8.9	682	10.9^bd^	460	6.4^bd^	387	8.8^ c^^d^	168	4.1^bd^

Association of educational attainment with the prevalence of ever smoking

The overall prevalence of ever smoking in the full sample was 41.6% among women and 53.6% among men. The overall prevalence across racial/ethnic groups ranged from 12.4% to 44.6% among women and 29.6% to 57.8% among men, with Chinese women and men having the lowest prevalence and Black and White women and Black and Filipino men having the highest prevalence (Table 2). The prevalence of ever smoking was significantly higher at the three lower levels of education compared to the college graduate level among men and women in all racial/ethnic groups and the full sample, with the exception of non-high school graduate Chinese women (Table 3). Compared to college graduates, the prevalence of ever smoking was 21% to 57% higher among women and 23% to 67% higher among men who were non-high school graduates, 32% to two-fold higher among women and 22% to 65% higher among men who were high school graduates, and 25% to two-fold higher among women and 21% to 50% higher among men with some college. The largest difference was seen among Filipina women, where the prevalence of ever smoking among high school graduates and those with some college was double that of college graduates. A step gradient relationship was seen for White women and a non-step gradient relationship for the full sample, White men, and Black adults. The prevalence of ever smoking did not significantly differ across the three lower levels of education among Latina women and Latino, Filipino, and Chinese men. Among Filipina and Chinese women, the prevalence of ever smoking was similar at the high school graduate and some college levels but lower than both of these levels at the non-high school graduate level.

**Table 3:** Comparison of the prevalence of ever smoking at four levels of education between women and men aged 65-79 years. %: age-standardized prevalence among adults with this level of education; in the "Full sample" column, the age-standardization model prevalence additionally controlled for racial/ethnic group; aPR: adjusted prevalence ratio; CI: confidence interval; GED: high school diploma equivalent; AD: associate (community college) degree; College graduate: ≥ bachelor’s degree. ^a^ aPRs in this column compare the prevalence at the lower three levels of education to the prevalence at the ≥ bachelor’s degree level. An aPR > 1.00 indicates a higher prevalence at the lower level of education compared to the prevalence at the college graduate level, while an aPR < 1.00 indicates a lower prevalence compared to that at the college graduate level. An aPR with a 95% CI that does not include 1.00 is statistically significant at p < 0.05. ^b^ Prevalence among adults at this level of education significantly (p < 0.05) differs from prevalence among adults at the next higher level based on an aPR that adjusted for age group (65-69, 70-74, 75-79 years); for the Full Sample, the aPRs adjusted for age group and racial/ethnic group. ^c^ The prevalence among adults at this level of education significantly (p < 0.05) differs from the prevalence among college graduates after adjusting for age group; for the "Full sample," the aPR adjusted for age group and racial/ethnic group. ^d^ The prevalence among adults in this racial/ethnic group significantly (p < 0.05) differs from the prevalence among White adults with the same level of education after adjusting for age group. ^e^ The prevalence among Filipino adults significantly (p < 0.05) differs from the prevalence among Chinese adults with the same level of education after adjusting for age group. ^f^ The prevalence among women significantly (p < 0.05) differs from the prevalence among men at the same level of education and of the same racial/ethnic group; for the "Full sample," the aPR testing for sex differences adjusted for age group and racial/ethnic group.

Level of education	Full sample		White		Black		Latino		Filipino		Chinese	
	%	aPR ^a^ (95% CI)	%	aPR ^a^ (95% CI)	%	aPR ^a^ (95% CI)	%	aPR ^a^ (95% CI)	%	aPR ^a^ (95% CI)	%	aPR ^a^ (95% CI)
Women												
Non-high school graduate	49.9	1.36^ c ^(1.31-1.40)	54.6^ b^	1.41^ c ^(1.36-1.47)	53.6	1.43^ c ^(1.30-1.57)	32.4^ bd^	1.21^ c ^(1.09-1.35)	15.1^ bde^	1.57^ c ^(1.23-2.00)	7.5^ bd^	0.77 (0.50-1.19)
High school graduate/GED	49.4^ b^	1.34^ c ^(1.31-1.37	50.1^ b^	1.32^ c ^(1.29-1.35)	50.6^ b^	1.35^ c ^(1.26-1.45)	36.2^d^	1.35^ c ^(1.23-1.49)	21.8^ de^	2.20^ c ^(1.81-2.67)	15.4^ d^	1.49^ c ^(1.16-1.90)
Some college/AD	46.8^ b^	1.26^ c ^(1.24-1.29)	46.7^ b^	1.25^ c ^(1.22-1.27)	46.4^ b^	1.25^ c ^(1.18-1.32)	34.0^ bd^	1.27^ c ^(1.16-1.39)	20.8^ bde^	2.13^ c ^(1.84-2.47)	17.1^ bd^	1.67^ c ^(1.10-2.00)
College graduate	37.9	(reference)	37.7	(reference)	37.5	(reference)	26.6^ d^	(reference)	9.7^ d^	(reference)	10.3^ d^	(reference)
Men												
Non-high school graduate	65.5^ f^	1.50^ c ^(1.46-1.54)	67.2^ f^	1.54^ c ^1.49-1.59)	74.9^ bdf^	1.53^ c ^(1.42-1.65)	57.4^ df^	1.33^ c ^(1.24-1.43)	64.6^ ef^	1.23^ c ^(1.11-1.36)	41.5^ df^	1.67^ c ^(1.40-1.99)
High school graduate/GED	64.8^ bf^	1.48^ c ^(1.45-1.50)	66.4^ bf^	1.53^ c ^(1.50-1.56)	63.2^ df^	1.31^ c ^(1.23-1.40)	57.0^ df^	1.31^ c ^(1.23-1.40)	64.2^ ef^	1.22^ c ^(1.13-1.32)	40.5^ df^	1.65^ c ^(1.43-1.90)
Some college/AD	60.3^ bf^	1.39^ c ^(1.37-1.41)	60.9^ bf^	1.41^ c ^(1.39-1.44)	61.0^ bf^	1.27^ c ^(1.21-1.34)	54.8^ bdf^	1.27^ c ^(1.20-1.35)	63.3^ bdef^	1.21^ c ^(1.14-1.28)	36.8^ bdf^	1.50^ c ^(1.35-1.66)
College graduate	43.8^ e^	(reference)	43.1^ e^	(reference)	48.1^ de^	(reference)	43.4^ e^	(reference)	52.4^ def^	(reference)	24.8^ df^	(reference)

Compared to White women, Latina, Filipina, and Chinese women were less likely to be ever smokers at all four levels of education, with Black women not significantly different from White women at any level of education (Tables 3, 4). Compared to White men, the prevalence of ever smoking was lower among Chinese men at all levels and Latino men at the lower three levels of education and higher among Filipino and Black men at the college graduate level. Compared to Chinese adults, Filipino men at all levels of education and women non-college graduates were more likely to be ever smokers. In all racial/ethnic groups, men were more likely to be ever smokers than women at all levels of education (Tables 3, 5).

**Table 4:** Comparison of the prevalence of ever smoking among racial/ethnic groups within the same level of education between women and men aged 65-79 years. %: age-standardized prevalence among adults with this level of education; aPR: adjusted prevalence ratio; CI: confidence interval; GED: high school diploma equivalent; AD: associate (community college) degree; College graduate: ≥ bachelor’s degree. ^a^ aPRs in this column compare the prevalence within this level of education between adults in this racial/ethnic group and the White group after adjusting for the age group. An aPR > 1.00 indicates a significantly higher prevalence and an aPR < 1.00 indicates a significantly lower prevalence in this racial/ethnic group compared to White adults if the 95% CI does not include 1.00. An aPR with a 95% CI that does not include 1.00 indicates that the between-group difference in the prevalence was statistically significant at p < 0.05 after adjusting for age group. ^b^ Meaningful difference in the prevalence at this level of education between adults in this racial/ethnic group compared to White adults based on a ≥ 1 percentage point difference between subgroups and an aPR that was statistically significant at p < 0.05.

Level of education	White		Black		Latino		Filipino		Chinese	
	%	aPR^ a^ (95% CI)	%	aPR^ a^ (95% CI)	%	aPR^ a^ (95% CI)	%	aPR^ a^ (95% CI)	%	aPR^ a^ (95% CI)
Women										
Non-high school graduate	54.6	(reference)	53.6	0.99 (0.90-1.08)	32.4 ^b^	0.60 (0.55-0.65)	15.1^ b^	0.27 (0.22-0.34)	7.5^ b^	0.14 (0.09-0.22)
High school graduate/GED	50.1	(reference)	50.6	1.01 (0.96-1.07)	36.2 ^b^	0.73 (0.68-0.77)	21.8^ b^	0.42 (0.36-049)	15.4^ b^	0.30 (0.24-0.37)
Some college/AD	46.7	(reference)	46.4	1.00 (0.96-1.03)	34.0^ b^	0.73 (0.69-0.77)	20.8^ b^	0.44 (0.40-0.50)	17.1^ b^	0.36 (0.31-0.41)
College graduate	37.7	(reference)	37.5	1.00 (0.95-1.05)	26.6^ b^	0.72 (0.67-0.78)	9.7^ b^	0.26 (0.23-0.29)	10.3^b^	0.27 (0.24-0.30)
Men										
Non-high school graduate	67.2	(reference)	74.9^ b^	1.09 (1.03-1.17)	57.4^ b^	0.86 (0.81-0.91)	64.6	0.96 (0.87-1.06)	41.5^ b^	0.61 (0.52-0.72)
High school graduate/GED	66.4	(reference)	63.2^ b^	0.95 (0.90-0.99)	57.0^ b^	0.86 (0.82-0.90)	64.2	0.96 (0.90-1.03)	40.5^ b^	0.61 (0.53-0.69)
Some college/AD	60.9	(reference)	61.0	1.00 (0.97-1.04)	54.8^ b^	0.90 (0.87-0.94)	63.3^ b^	1.05 (1.01-1.10)	36.8^ b^	0.60 (0.55-0.66)
College graduate	43.1	(reference)	48.1^ b^	1.12 (1.07-1.17)	43.4	1.01 (0.96-1.06)	52.4^ b^	1.21 (1.17-1.26)	24.8^ b^	0.57 (0.54-0.61)

**Table 5:** Sex differences in the prevalence of ever smoking within four levels of education by race and ethnicity in adults aged 65-79 years. %: age-standardized prevalence among adults with this level of education; aPR: adjusted prevalence ratio; CI: confidence interval; GED: high school diploma equivalent; AD: associate (community college) degree; College graduate: ≥ bachelor’s degree. ^a^ aPRs in this column compare the prevalence within this level of education between men and women after adjusting for age group. An aPR > 1.00 indicates a higher prevalence and an aPR < 1.00 indicates a lower prevalence among men than women if the 95% CI does not include 1.00. An aPR with a 95% CI that does not include 1.00 indicates that men were significantly different from women within the same level of education at p < 0.05 after adjusting for age group. ^b^ Meaningful difference in the prevalence at this level of education between men and women in this racial/ethnic group based on a ≥ 1 percentage point difference between subgroups and an aPR that is statistically significant at p < 0.05.

Level of education	White		Black		Latino		Filipino		Chinese	
	%	aPR^ a^ (95% CI)	%	aPR^ a^ (95% CI)	%	aPR^ a ^(95% CI)	%	aPR^ a ^(95% CI)	%	aPR^ a^ (95% CI)
Women										
Non-high school graduate	54.6	(reference)	53.6	(reference)	32.4	(reference)	15.1	(reference)	7.5	(reference)
High school graduate/GED	50.1	(reference)	50.6	(reference)	36.2	(reference)	21.8	(reference)	15.4	(reference)
Some college/AD	46.7	(reference)	46.4	(reference)	34.0	(reference)	20.8	(reference)	17.1	(reference)
College graduate	37.7	(reference)	37.5	(reference)	26.6	(reference)	9.7	(reference)	10.3	(reference)
Men										
Non-high school graduate	67.2^ b^	1.26 (1.20-1.32)	74.9^ b^	1.39 (1.26-1.53)	57.4^ b^	1.80 (1.64-1.97)	64.6^ b^	4.31 (3.39-5.47)	41.5^ b^	5.35 (3.41-8.39)
High school graduate/GED	66.4^ b ^	1.34 (1.31-1.37)	63.2^ b^	1.25 (1.17-1.35)	57.0^ b^	1.57 (1.47-1.69)	64.2^ b ^	3.06 (2.57-3.65)	40.5^ b^	2.69 (2.11-3.43)
Some college/AD	60.9^ b^	1.30 (1.28-1.32)	61.0^ b^	1.31 (1.25-1.37)	54.8^ b^	1.61 (1.51-1.71)	63.3^ b^	3.06 (2.72-3.43)	36.8^ b^	2.19 (1.87-2.56)
College graduate	43.1^ b^	1.14 (1.12-1.16)	48.1^ b^	1.28 (1.20-1.36)	43.4^ b^	1.60 (1.46-1.75)	52.4^ b^	5.38 (4.83-6.00)	24.8^ b^	2.44 (2.13-2.79)

Association of educational attainment with the prevalence of current smoking

The overall prevalence of current smoking in the full sample was 4.8% among women and 5.7% among men. Across racial/ethnic groups, the overall prevalence ranged from 1.5% to 8.1% among women and 3.0% to 9.4% among men, with Chinese adults having the lowest prevalence and Black adults having the highest prevalence (Table 2). With the exception of Chinese women, the prevalence of current smoking among women and men in the full sample and all five racial/ethnic groups was significantly higher at all levels of education compared to prevalence at the college graduate level, with aPRs indicating a two-fold to three-fold higher prevalence among non-high school graduates and an approximately two-fold higher prevalence among high school graduates and adults with some college among most racial/ethnic subgroups (Table 6). A step gradient relationship of educational attainment with current smoking was seen for men and women in the full sample and White group, and a non-step gradient relationship was seen in Black and Latino adults and Chinese men. The prevalence of current smoking was similar across all three lower levels of education among Filipino men and women and across all four levels of education for Chinese women. Notably, the prevalence of current smoking was very low (range = 1-3%) at all levels of education among Filipina and Chinese women.

**Table 6:** Comparison of the prevalence of current smoking at four levels of education between women and men aged 65-79 years. %: age-standardized prevalence among adults with this level of education; in the "Full sample" column, the age-standardization model prevalence additionally controlled for racial/ethnic group; aPR: adjusted prevalence ratio; CI: confidence interval; GED: high school diploma equivalent; AD: associate degree; College graduate: ≥ bachelor’s degree. ^a^ aPRs in this column compare the prevalence at the lower three levels of education to the prevalence at the ≥ bachelor’s degree level. An aPR > 1.00 indicates a higher prevalence at the lower level of education compared to the prevalence at the college graduate level, while an aPR < 1.00 indicates a lower prevalence compared to that at the college graduate level. An aPR with a 95% CI that does not include 1.00 is statistically significant at p < 0.05. ^b^ The prevalence among adults at this level of education significantly (p < 0.05) differs from the prevalence among adults at the next higher level based on an aPR that adjusted for age group (65-69, 70-74, 75-79 years); for the "Full sample," the aPRs adjusted for age group and racial/ethnic group. ^c^ The prevalence among adults at this level of education significantly (p < 0.05) differs from the prevalence among college graduates after adjusting for age group; for the "Full sample," the aPR adjusted for age group and racial/ethnic group. ^d^ The prevalence among adults in this racial/ethnic group significantly (p < 0.05) differs from the prevalence among White adults with the same level of education after adjusting for age group. ^e^ The prevalence among Filipino adults significantly (p < 0.05) differs from the prevalence among Chinese adults with the same level of education after adjusting for age group. ^f^ The prevalence among women significantly (p < 0.05) differs from the prevalence among men at the same level of education and of the same racial/ethnic group; for the "Full sample," the aPR testing for sex differences adjusted for age group and racial/ethnic group.

Level of education	Full sample		White		Black		Latino		Filipino		Chinese	
	%	aPR^ a^ (95% CI)	%	aPR^ a^ (95% CI)	%	aPR^ a^ (95% CI)	%	aPR^ a^ (95% CI)	%	aPR^ a^ (95% CI)	%	aPR^ a^ (95% CI)
Women												
Non-high school graduate	9.4^ b^	3.10^ c ^(2.78-3.45)	10.7^ b^	3.28^ c^ (2.87-3.75)	13.7	2.74^ c^ (2.07-3.61)	6.6^ bd^	2.88^ c^ (2.03-4.07)	3.4^ d^	2.08^ c^ (1.21-3.57)	2.1^ d^	2.04 (0.85-4.91)
High school graduate/GED	7.8^ b^	2.43^ c ^(2.26-2.61)	8.0^ b^	2.50^ c^ (2.31-2.71)	11.2^ bd^	2.27^ c^ (1.83-2.83)	4.6^ d^	1.91^ c^ (1.36-2.69)	3.1^ d^	1.65(0.94-2.88)	3.6^ d^	3.04^ c ^(1.68-5.52)
Some college/AD	6.1^ b^	1.89^ c ^(1.77-2.02)	6.1^ b^	1.92^ c^ (1.79-2.07)	8.7^ bd^	1.78^ c^ (1.48-2.15)	4.0^ bd^	1.68^ c^ (1.20-2.34)	3.4^ bde^	1.90^ c^ (1.30-2.80)	1.5^ d^	1.31 (0.72-2.37)
College graduate	3.3	(reference)	3.2	(reference)	4.9^ d^	(reference)	2.4	(reference)	1.7^ d^	(reference)	1.1^ d^	(reference)
Men												
Non-high school graduate	11.4^ bf^	3.17^ c ^(2.85-3.53)	12.8^ bf^	3.59^ c^ (3.14-4.10)	16.8	2.54^ c^ (1.91-3.40)	7.8^ d^	2.53^ c^ (1.84-3.48)	8.4^ f^	2.14^ c^ (1.34-3.39)	7.6^ df^	3.64^ c^ (2.14-6.17)
High school graduate/GED	9.0^ bf^	2.49^ c ^(2.30-2.69)	9.2^ bf^	2.60^ c^ (2.38-2.85)	12.1^ d^	1.94^ c^ (1.54-2.46)	7.1^ bdf^	2.27^ c^ (1.67-3.09)	8.7^ f^	2.23^ c^ (1.59-3.14)	5.5^ d^	2.46^ c^ (1.49-4.06)
Some college/AD	7.2^ bf^	2.00^ c^ (1.88-2.13	7.2^ bf^	2.06^ c^ (1.91-2.20)	10.1^ bd^	1.64^ c^ (1.35-2.00)	5.8^ bdf^	1.91^ c^ (1.43-2.55)	7.5^ bef^	1.88^ c^ (1.43-2.49)	3.9^ bdf^	1.88^ c^ (1.28-2.78)
College graduate	3.6	(reference)	3.5	(reference)	6.2^ df^	(reference)	3.1	(reference)	4.0^ ef^	(reference)	2.1^ df^	(reference)

An examination of racial/ethnic differences within the same level of education found that at the high school graduate and some college levels, Black adults were more likely and Latino and Chinese adults and Filipina women were less likely than their White counterparts to be current smokers. At the college graduate level, Black adults were also more likely than White adults to be current smokers (Tables 6, 7). Compared to Chinese adults, Filipino men at ≥ some college levels and women at some college level were more likely to be current smokers. Sex differences within the level of education were also seen, with White, Latino, and Filipino men more likely than women to be current smokers at the three lower levels of education and Black, Filipino, and Chinese men more likely to be current smokers at the college graduate level (Tables 6, 8).

**Table 7:** Comparison of the prevalence of current smoking among racial/ethnic groups within the same level of education between women and men aged 65-79 years. %: age-standardized prevalence among adults with this level of education; aPR: adjusted prevalence ratio; CI: confidence interval; GED: high school diploma equivalent; AD: associate (community college) degree; College graduate: ≥ bachelor’s degree. ^a^ aPRs in this column compare the prevalence within this level of education between adults in this racial/ethnic group and the White group after adjusting for the age group. An aPR > 1.00 indicates a significantly higher prevalence and an aPR < 1.00 indicates a significantly lower prevalence in this racial/ethnic group compared to White adults if the 95% CI does not include 1.00. An aPR with a 95% CI that does not include 1.00 indicates that the between-group difference in the prevalence was statistically significant at p < 0.05 after adjusting for age group. ^b^ Meaningful difference in the prevalence at this level of education between adults in this racial/ethnic group compared to White adults based on a ≥ 1 percentage point difference between subgroups and an aPR that is statistically significant at p < 0.05.

Level of education	White		Black		Latino		Filipino		Chinese	
	%	aPR^ a^ (95% CI)	%	aPR^ a^ (95% CI)	%	aPR^ a^ (95% CI)	%	aPR^ a^ (95% CI)	%	aPR^ a^ (95% CI)
Women										
Non-high school graduate	10.7	(reference)	13.7	1.26 (0.98-1.63)	6.6^ b^	0.64 (0.50-0.81)	3.4^ b^	0.35 (0.21-0.58)	2.1^ b^	0.22 (0.10-0.48)
High school graduate/GED	8.0	(reference)	11.2^ b^	1.38 (1.17-1.62)	4.6^ b^	0.57 (0.46-0.69)	3.1^ b^	0.36 (0.22-0.60)	3.6^ b^	0.42 (0.27-0.68)
Some college/AD	6.1	(reference)	8.7^ b^	1.43 (1.28-1.59)	4.0^ b^	0.67 (0.55-0.80)	3.4^ b^	0.55 (0.41-0.74)	1.5^ b^	0.24 (0.15-0.39)
College graduate	3.2	(reference)	4.9^ b^	1.54 (1.30-1.82)	2.4	0.77 (0.58-1.02)	1.7^ b^	0.55 (0.32-0.71)	1.1^ b^	0.35 (0.25-0.51)
Men										
Non-high school graduate	12.8	(reference)	16.8	1.24 (0.96-1.62)	7.8^ b^	0.61 (0.48-0.78)	8.4	0.68 (0.33-1.04)	7.6^ b^	0.60 (0.37-0.98)
High school graduate/GED	9.2	(reference)	12.1^ b^	1.31(1.09-1.57)	7.1^ b^	0.76 (0.62-0.93)	8.7	0.97 (0.72-1.29)	5.5^ b^	0.59 (0.38-0.91)
Some college/AD	7.2	(reference)	10.1^ b^	1.41 (1.25-1.59)	5.8^ b^	0.82 (0.69-0.96)	7.5	1.04 (0.85-1.27)	3.9^ b^	0.57 (0.42-0.77)
College graduate	3.5	(reference)	6.2^ b^	1.77 (1.49-2.10)	3.1	0.88 (0.69-1.13)	4.0	1.13 (0.92-1.39)	2.1^ b^	0.62 (0.49-0.80)

**Table 8:** Sex differences in the prevalence of current smoking within four levels of education, by race and ethnicity, among adults aged 65-79 years. %: age-standardized prevalence among adults with this level of education; aPR: adjusted prevalence ratio; CI: confidence interval; GED: high school diploma equivalent; AD: associate (community college) degree; College graduate: ≥ bachelor’s degree. ^a^ aPRs in this column compare the prevalence within this level of education between men and women after adjusting for age group. An aPR > 1.00 indicates a higher prevalence and an aPR < 1.00 indicates a lower prevalence among men than women if the 95% CI does not include 1.00. An aPR with a 95% CI that does not include 1.00 indicates that men were significantly different from women within the same level of education at p < 0.05 after adjusting for age group. ^b^ Meaningful difference in the prevalence at this level of education between men and women in this racial/ethnic group based on a ≥ 1 percentage point difference between subgroups and an aPR that is statistically significant at p < 0.05.

Level of education	White		Black		Latino		Filipino		Chinese	
	%	aPR^ a^ (95% CI)	%	aPR^ a^ (95% CI)	%	aPR^ a^ (95% CI)	%	aPR^ a^ (95% CI)	%	aPR^ a^ (95% CI)
Women										
Non-high school graduate	10.7	(reference)	13.7	(reference)	6.6	(reference)	3.4	(reference)	2.1	(reference)
High school graduate/GED	8.0	(reference)	11.2	(reference)	4.6	(reference)	3.1	(reference)	3.6	(reference)
Some college/AD	6.1	(reference)	8.7	(reference)	4.0	(reference)	3.4	(reference)	1.5	(reference)
College graduate	3.2	(reference)	4.9	(reference)	2.4	(reference)	1.7	(reference)	1.1	(reference)
Men										
Non-high school graduate	12.8	1.17 (0.85-1.62)	16.8^ b^	1.21 (1.02-1.43)	7.8	1.17 (0.88-1.56)	8.4^ b^	2.38 (1.27-4.45)	7.6^ b^	3.44 (1.37-8.58)
High school graduate/GED	9.2	1.08 (0.86-1.36)	12.1^ b^	1.15 (1.05-1.26)	7.1^ b^	1.56 (1.19-2.04)	8.7^ b ^	3.04 (1.71-5.40)	5.5	1.58 (0.84-2.98)
Some college/AD	7.2^ b^	1.16 (1.01-1.35)	10.1^ b^	1.18 (1.10-1.26)	5.8^ b^	1.45 (1.14-1.84)	7.5^ b^	2.24 (1.58-3.18)	3.9^ b^	2.76 (1.58-4.84)
College graduate	3.5	1.26 (1.01-1.58)	6.2^ b^	1.10 (1.02-1.19)	3.1	1.25 (0.87-1.80)	4.0^ b^	2.28 (1.66-3.13)	2.1^ b^	1.93 (1.25-2.97)

Not shown is that among ever smokers, Black adults were less likely than White and Latino adults and Filipino men at high school graduate, some college, and college graduate levels to have quit smoking by 2016.

Association of educational attainment with the prevalence of diabetes

The overall prevalence of diabetes in the full sample was 15.2% among women and 21.9% among men. Across racial/ethnic groups, the overall prevalence ranged from 15.2% to 42.6% among women and 22.0% to 50.5% among men, with White women and men having the lowest prevalence and Filipino women and men having the highest prevalence (Table 2). The prevalence of diabetes was significantly higher at the three lower levels of education compared to the college graduate level among women in the full sample and all racial/ethnic groups and among White, Black, and Latino men (Table 9). Specifically, compared to college graduates, the prevalence of diabetes was 20% to two-fold higher among women and 31% to 85% higher among men who were non-high school graduates, 11% to 87% higher among women and 15% to 62% higher among men who were high school graduates, and 9% to 58% higher among women and 11% to 44% higher among men with some college. The largest differences were seen among White adults, with an approximately two-fold higher prevalence of diabetes among non-high school graduates and a 60% to 80% higher prevalence among high school graduates compared to college graduates. A step gradient relationship was seen for the full sample and White and Latino adults, and a non-step gradient relationship was seen for Black and Chinese adults and Filipina women. Diabetes prevalence did not significantly differ across levels of education among Filipino men.

**Table 9:** Comparison of the prevalence of diabetes at four levels of education between women and men aged 65-79 years. %: age-standardized prevalence among adults with this level of education; In the "Full sample" column, the age-standardization model prevalence additionally controlled for racial/ethnic group; aPR: adjusted prevalence ratio; CI: confidence interval; GED: high school diploma equivalent; AD: associate degree; College graduate: ≥ bachelor’s degree. ^a^ aPRs in this column compare the prevalence at the lower three levels of education to the prevalence at the ≥ bachelor’s degree level. An aPR > 1.00 indicates a higher prevalence at the lower level of education compared to the prevalence at the college graduate level, while an aPR < 1.00 indicates a lower prevalence compared to that at the college graduate level. An aPR with a 95% CI that does not include 1.00 is statistically significant at p < 0.05. ^b^ The prevalence among adults at this level of education significantly (p < 0.05) differs from the prevalence among adults at the next higher level based on an aPR that adjusted for age group (65-69, 70-74, 75-79 years); for the "Full sample," the aPRs adjusted for age group and racial/ethnic group. ^c^ The prevalence among adults at this level of education significantly (p < 0.05) differs from the prevalence among college graduates after adjusting for age group; for the "Full sample," the aPR adjusted for age group and racial/ethnic group. ^d^ The prevalence among adults in this racial/ethnic group significantly (p < 0.05) differs from the prevalence among White adults with the same level of education after adjusting for age group. ^e^ The prevalence among Filipino adults significantly (p < 0.05) differs from the prevalence among Chinese adults with the same level of education after adjusting for age group. ^f^ The prevalence among women significantly (p < 0.05) differs from the prevalence among men at the same level of education and of the same racial/ethnic group; for the "Full sample," the aPR testing for sex differences adjusted for age group and racial/ethnic group.

Level of education	Full sample		White		Black		Latino		Filipino		Chinese	
	%	aPR^ a^ (95% CI)	%	aPR^ a^ (95% CI)	%	aPR^ a^ (95% CI)	%	aPR^ a^ (95% CI)	%	aPR^ a^ (95% CI)	%	aPR^ a^ (95% CI)
Women												
Non-high school graduate	24.1^ b^	1.76^ c^ (1.68-1.84)	24.0^ b^	2.18^ c^ (2.02-2.35)	39.8^ d^	1.42^ c^ (1.26-1.59)	40.4^ bd^	1.63^ c^ (1.47-1.80)	49.6^ de^	1.20^ c^ (1.09-1.33)	28.1	1.54^ c^ (1.24-1.91)
High school graduate/GED	20.4^ b^	1.62^ c^ (1.57-1.67)	20.6^ b^	1.87^ c^ (1.79-1.95)	38.0^ d^	1.33^ c^ (1.22-1.46)	34.4^ bd^	1.37^ c^ (1.24-1.51)	45.2^ de^	1.11^ c^ (1.01-1.23)	25.1^ d^	1.42^ c^ (1.19-1.68)
Some college/AD	17.3^ b^	1.42^ c^ (1.38-1.46)	17.4^ b^	1.58^ c^ (1.52-1.64)	35.4^ bd^	1.24^ c^ (1.15-1.33)	31.0^ bd^	1.24^ c^ (1.13-1.37)	44.3^ de^	1.09^ c^ (1.01-1.17)	23.0^ bd^	1.29^ c^ (1.12-1.48)
College graduate	11.2	(reference)	11.1	(reference)	28.6^ d^	(reference)	25.2^ d^	(reference)	40.7^ de^	(reference)	18.4^ d^	(reference)
Men												
Non-high school graduate	31.9^ bf^	1.56^ c^ (1.50-1.63)	33.6^ bf^	1.85^ c^ (1.73-1.98)	45.9^ df^	1.32^ c^ (1.17-1.49)	45.9^ bdf^	1.43^ c^ (1.31-1.57)	50.8^ de^	1.00 (0.88-1.13)	35.4	1.31^ c^ (1.08-1.60)
High school graduate/GED	28.2^ bf^	1.44^ c^ (1.40-1.49)	29.0^ bf^	1.62^ c^ (1.55-1.68)	42.1^ bd^	1.23^ c^ (1.12-1.34)	41.5^ bdf^	1.30^ c^ (1.19-1.42)	48.9^ df^	0.94 (0.86-1.04)	29.8	1.15 (0.97-1.36)
Some college/AD	25.3^ bf^	1.32^ c^ (1.28-1.35)	25.8^ bf^	1.44^ c^ (1.39-1.49)	38.2^ bdf^	1.11^ c^ (1.03-1.20)	39.3^ bdf^	1.22^ c^ (1.13-1.32)	49.6^ def^	0.96 (0.90-1.03)	29.4^ df^	1.12^ c^ (1.01-1.26)
College graduate	18.4^ f^	(reference)	17.9^ f^	(reference)	34.4^ df^	(reference)	32.0^ df^	(reference)	51.4^ def^	(reference)	27.0^ df^	(reference)

Compared to White adults, the prevalence of diabetes was higher among Black, Latino, and Filipino women at all four levels of education, Chinese women at the upper three levels, and Chinese men at the upper two levels (Tables 9, 10). Filipino adults were more likely to have diabetes than Chinese adults at all levels of education. Among White, Black, and Latino adults, men were more likely to have diabetes than women at all levels of education, whereas, among Filipino and Chinese adults, a similar sex difference was only seen at some college and college graduate levels (Tables 9, 11).

**Table 10:** Comparison of the prevalence of diabetes among racial/ethnic group within the same level of education between women and men aged 65-79 years. %: age-standardized prevalence among adults with this level of education; aPR: adjusted prevalence ratio; CI: confidence interval; GED: high school diploma equivalent; AD: associate (community college) degree; College graduate: ≥ bachelor’s degree. ^a^ aPRs in this column compare the prevalence within this level of education between adults in this racial/ethnic group and the White group after adjusting for the age group. An aPR > 1.00 indicates a significantly higher prevalence and an aPR < 1.00 indicates a significantly lower prevalence in this racial/ethnic group compared to White adults if the 95% CI does not include 1.00. An aPR with a 95% CI that does not include 1.00 indicates that the between-group difference in the prevalence was statistically significant at p < 0.05 after adjusting for age group. ^b^ Meaningful difference in the prevalence at this level of education between adults in this racial/ethnic group compared to White adults based on a ≥ 1 percentage point difference between subgroups and an aPR that is statistically significant at p < 0.05.

Level of education	White		Black		Latino		Filipino		Chinese	
	%	aPR^ a^ (95% CI)	%	aPR^ a^ (95% CI)	%	aPR^ a^ (95% CI)	%	aPR^ a^ (95% CI)	%	aPR^ a^ (95% CI)
Women										
Non-high school graduate	24.0	(reference)	39.8^ b^	1.70 (1.51-1.92)	40.4^ b^	1.67 (1.52-1.84)	49.6^ b^	2.01 (1.79-2.25)	28.1	1.15 (0.94-1.42)
High school graduate/GED	20.6	(reference)	38.0^ b^	1.85 (1.72-1.99)	34.4^ b^	1.65 (1.54-1.77)	45.2^ b^	2.17 (1.97-2.39)	25.1^ b^	1.24 (1.06-1.44)
Some college/AD	17.4	(reference)	35.4^ b^	2.03 (1.94-2.13)	31.0^ b^	1.78 (1.68-1.90)	44.3^ b^	2.55 (2.38-2.72)	23.0^ b^	1.32 (1.19-1.48)
College graduate	11.1	(reference)	28.6^ b^	2.58 (2.42-2.76)	25.2^ b^	2.26 (2.08-2.46)	40.7^ b^	3.68 (3.51-3.87)	18.4^ b^	1.62 (1.48-1.77)
Men										
Non-high school graduate	33.6	(reference)	45.9^ b^	1.35 (1.20-1.53)	45.9^ b^	1.37 (126-1.50)	50.8^ b^	1.53 (1.33-175)	35.4	1.04 (0.86-1.27)
High school graduate/GED	29.0	(reference)	42.1^ b^	1.44 (1.33-1.56)	41.5^ b^	1.43 (1.35-1.53)	48.9^ b^	1.67 (1.52-1.83)	29.8	1.04 (0.89-1.23)
Some college/AD	25.8	(reference)	38.2^ b^	1.49 (1.41-1.57)	39.3^ b^	1.53 (1.44-1.61)	49.6^ b^	1.93 (1.82-2.05)	29.4^ b^	1.15 (1.04-1.28)
College graduate	17.9	(reference)	34.4^ b^	1.92 (1.81-2.04)	32.0^ b^	1.80 (1.68-1.92)	51.4^ b^	2.86 (2.74-2.99)	27.0^ b^	1.49 (1.39-1.59)

**Table 11:** Sex differences in the prevalence of diabetes within four levels of education, by race and ethnicity, among adults aged 65-79 years. %: age-standardized prevalence among adults with this level of education; aPR: adjusted prevalence ratio; CI: confidence interval; GED: high school diploma equivalent; AD: associate (community college) degree; College graduate: ≥ bachelor’s degree. ^a^ aPRs in this column compare the prevalence within this level of education between men and women after adjusting for age group. An aPR > 1.00 indicates a higher prevalence and an aPR < 1.00 indicates a lower prevalence among men than women if the 95% CI does not include 1.00. An aPR with a 95% CI that does not include 1.00 indicates that men were significantly different from women within the same level of education at p < 0.05 after adjusting for age group. ^b^ Meaningful difference in the prevalence at this level of education between men and women in this racial/ethnic group based on a ≥ 1 percentage point difference between subgroups and an aPR that is statistically significant at p < 0.05.

Level of education	White		Black		Latino		Filipino		Chinese	
	%	aPR^ a ^(95% CI)	%	aPR^ a^ (95% CI)	%	aPR^ a^ (95% CI)	%	aPR^ a^ (95% CI)	%	aPR^ a^ (95% CI)
Women										
Non-high school graduate	24.0	(reference)	39.8	(reference)	40.4	(reference)	49.6	(reference)	28.1	(reference)
High school graduate/GED	20.6	(reference)	38.0	(reference)	34.4	(reference)	45.2	(reference)	25.1	(reference)
Some college/AD	17.4	(reference)	35.4	(reference)	31.0	(reference)	44.3	(reference)	23.0	(reference)
College graduate	11.1	(reference)	28.6	(reference)	25.2	(reference)	40.7	(reference)	18.4	(reference)
Men										
Non-high school graduate	33.6^ b^	1.40 (1.27-1.53)	45.9	1.11 (0.96-1.29)	45.9^ b^	1.14 (1.05-1.25)	50.8	1.04 (0.89-1.21)	35.4	1.25 (0.95-1.64)
High school graduate/GED	29.0^ b^	1.42 (1.35-1.48)	42.1	1.10 (1.00-1.22)	41.5^ b^	1.23 (1.13-1.33)	48.9	1.08 (0.95-1.22)	29.8	1.17 (0.94-1.46)
Some college/AD	25.8^ b^	1.48 (1.43-1.53)	38.2^ b^	1.08 (1.01-1.15)	39.3^ b^	1.27 (1.17-1.36)	49.6^ b^	1.12 (1.03-1.21)	29.4^ b^	1.28 (1.11-1.48)
College graduate	17.9^ b^	1.62 (1.56-1.68)	34.4^ b^	1.20 (1.11-1.30)	32.0^ b^	1.29 (1.16-1.42)	51.4^ b^	1.26 (1.19-1.34)	27.0^ b^	1.48 (1.33-1.64)

Association of educational attainment with the prevalence of hypertension

The overall prevalence of hypertension in the full sample was 54.5% among women and 59.4% among men. Across racial/ethnic groups, the prevalence ranged from 54.0% to 80.5% among women and 60.1% to 79.2% among men, with White and Chinese women and men having the lowest prevalence and Filipino women and men having the highest prevalence (Table 2). The prevalence of hypertension was significantly higher at the three lower levels of education among adults in the full sample and among White and Latino adults and Black women than among college graduates (Table 12). Among Chinese adults, the prevalence was higher among high school graduates (women only) and men and women with some college level than among college graduates. Compared to college graduates, the prevalence of hypertension was 8% to 38% higher among women and 10% to 27% higher among men who were non-high school graduates, 12% to 36% higher among women and 9% to 24% higher among men who were high school graduates, and 8% to 21% higher among women and 12% to 17% higher among men with some college. The largest differences were seen among White adults. A step gradient relationship was only seen for Latina women, and a non-step gradient relationship was seen for adults in the full sample and for White and Filipina women.

**Table 12:** Comparison of the prevalence of hypertension at four levels of education between women and men aged 65-79 years. %: age-standardized prevalence among adults with this level of education; in the "Full sample" column, the age-standardization model prevalence additionally controlled for racial/ethnic group; aPR: adjusted prevalence ratio; CI: confidence interval; GED: high school diploma equivalent; AD: associate degree; College graduate: ≥ bachelor’s degree. ^a^ aPRs in this column compare the prevalence at the lower three levels of education to the prevalence at the ≥ bachelor’s degree level. An aPR > 1.00 indicates a higher prevalence at the lower level of education compared to the prevalence at the college graduate level, while an aPR < 1.00 indicates a lower prevalence compared to that at the college graduate level. An aPR with a 95% CI that does not include 1.00 is statistically significant at p < 0.05. ^b^ The prevalence among adults at this level of education significantly (p < 0.05) differs from the prevalence among adults at the next higher level based on an aPR that adjusted for age group (65-69, 70-74, 75-79 years); for the "Full sample," the aPRs adjusted for age group and racial/ethnic group. ^c^ The prevalence among adults at this level of education significantly (p < 0.05) differs from the prevalence among college graduates after adjusting for age group; for the "Full sample," the aPR adjusted for age group and racial/ethnic group. ^d^ The prevalence among adults in this racial/ethnic group significantly (p < 0.05) differs from the prevalence among White adults with the same level of education after adjusting for age group. ^e^ The prevalence among Filipino adults significantly (p < 0.05) differs from the prevalence among Chinese adults with the same level of education after adjusting for age group. ^f^ The prevalence among women significantly (p < 0.05) differs from the prevalence among men at the same level of education and of the same racial/ethnic group; for the "Full sample," the aPR testing for sex differences adjusted for age group and racial/ethnic group.

Level of education	Full sample		White		Black		Latino		Filipino		Chinese	
	%	aPR^ a^ (95% CI)	%	aPR^ a^ (95% CI)	%	aPR^ a^ (95% CI)	%	aPR^ a^ (95% CI)	%	aPR^ a^ (95% CI)	%	aPR^ a^ (95% CI)
Women												
Non-high school graduate	63.0	1.29^ c^ (1.27-1.31)	64.0	1.38^ c^ (1.34-1.42)	85.6^ d^	1.14^ c^ (1.09-1.18)	72.5^ bd^	1.29^ c^ (1.22-1.35)	84.4^ de^	1.08^ c^ (1.04-1.12)	55.8^ d^	1.08 (0.96-1.21)
High school graduate/GED	62.8^ b^	1.30^ c^ (1.28-1.32)	63.7^ b^	1.36^ c^ (1.34-1.38)	85.0^ d^	1.13^ c^ (1.10-1.16)	68.9^ bd^	1.24^ c^ (1.18-1.30)	80.3^ de^	1.04 (0.99-1.08)	58.6^ d^	1.12^ c^ (1.03-1.22)
Some college/AD	57.9^ b^	1.21^ c^ (1.19-1.22)	58.4^ b^	1.26^ c^ (1.24-1.27)	81.7^ bd^	1.08^ c^ (1.06-1.11)	64.0^ bd^	1.15^ c^ (1.09-1.21)	80.5^ bde^	1.04 (1.01-1.07)	57.8^ b^	1.11^ c^ (1.04-1.18)
College graduate	47.5	(reference)	46.7	(reference)	75.5^ d^	(reference)	56.0^ d^	(reference)	77.7^ de^	(reference)	52.8^ d^	(reference)
Men												
Non-high school graduate	68.1^ f^	1.20^ c^ (1.17-1.22)	70.9^ f^	1.27^ c^ (1.23-1.31)	83.6^ d^	1.10^ c^ (1.05-1.16)	72.6	1.17^ c^ (1.12-1.23)	75.7^ ef^	0.94 (0.87-1.02)	62.8^ d^	1.06 (0.95-1.18)
High school graduate/GED	67.6^ bf^	1.20^ c^ (1.18-1.21)	68.5^ f^	1.24^ c^ (1.22-1.26)	82.2^ bd^	1.09^ c^ (1.05-1.13)	71.8^ df^	1.16^ c^ (1.11-1.22)	81.5^ de^	1.02 (0.98-1.07)	62.9^ d^	1.06 (0.97-1.15)
Some college/AD	64.1^ bf^	1.14^ c^ (1.13-1.15)	64.8^ bf^	1.17^ c^ (1.16-1.19)	77.6^ df^	1.03 (1.01-1.06)	69.1^ bdf^	1.12^ c^ (1.07-1.17)	78.3^ de^	0.98 (0.95-1.02)	63.9^ bf^	1.08^ c^ (1.02-1.15)
College graduate	55.9^ f^	(reference)	55.3^ f^	(reference)	75.4^ d^	(reference)	62.0^ df^	(reference)	79.7^ de^	(reference)	59.3^ df^	(reference)

The prevalence of hypertension was higher among Black, Latino, and Filipino adults than White adults at all four levels of education (Tables 12, 13). Hypertension prevalence among Chinese adults was lower than White adults among non-high school graduates and high school graduates, higher among college graduates, and similar to those with some college. Filipino adults were more likely to have hypertension than Chinese adults at all levels of education. Among White and Latino adults, men were more likely to have hypertension than women at all levels of education, with sex differences seen at some levels of education for Black and Chinese adults (Tables 12, 14).

**Table 13:** Comparison of the prevalence of hypertension among racial/ethnic groups within the same level of education between women and men aged 65-79 years. %: age-standardized prevalence among adults with this level of education; aPR: adjusted prevalence ratio; CI: confidence interval; GED: high school diploma equivalent; AD: associate (community college) degree; College graduate: ≥ bachelor’s degree. ^a^ aPRs in this column compare prevalence within the level of education between adults in this racial/ethnic group and the White group after adjusting for the age group. An aPR > 1.00 indicates a significantly higher prevalence and an aPR < 1.00 indicates a significantly lower prevalence in this racial/ethnic group compared to White adults if the 95% CI does not include 1.00. An aPR with a 95% CI that does not include 1.00 indicates that the between-group difference in the prevalence was statistically significant at p < 0.05 after adjusting for age group. ^b^ Meaningful difference in the prevalence at this level of education between adults in this racial/ethnic group compared to White adults based on a ≥ 1 percentage point difference between subgroups and an aPR that is statistically significant at p < 0.05.

Level of education	White		Black		Latino		Filipino		Chinese	
	%	aPR^ a^ (95% CI)	%	aPR^ a^ (95% CI)	%	aPR^ a^ (95% CI)	%	aPR^ a^ (95% CI)	%	aPR^ a^ (95% CI)
Women										
Non-high school graduate	64.0	(reference)	85.6^ b^	1.29 (1.24-1.35)	72.5^ b^	1.10 (1.06-1.14)	84.4^ b^	1.27 (1.22-1.33)	55.8^ b^	0.87 (0.78-0.97)
High school graduate/GED	63.7	(reference)	85.0^ b^	1.33 (1.29-1.36)	68.9^ b^	1.08 (1.04-1.11)	80.3^ b^	1.25 (1.20-1.31)	58.6^ b^	0.92 (0.85-0.99)
Some college/AD	58.4	(reference)	81.7^ b^	1.41 (1.38-1.43)	64.0^ b^	1.09 (1.06-1.13)	80.5^ b^	1.38 (1.34-1.42)	57.8^ b^	0.99 (0.94-1.04)
College graduate	46.7	(reference)	75.5^ b^	1.64 (1.60-1.68)	56.0^ b^	1.20 (1.15-1.25)	77.7^ b^	1.67 (1.64-1.71)	52.8^ b^	1.12 (1.07-1.16)
Men										
Non-high school graduate	70.9	(reference)	83.6^ b^	1.16 (1.11-1.22)	72.6	1.03 (0.99-1.08)	75.7	1.06 (0.98-1.14)	62.8^ b^	0.88 (0.79-0.98)
High school graduate/GED	68.5	(reference)	82.2^ b^	1.19 (1.15-1.23)	71.8^ b^	1.05 (1.02-1.09)	81.5^ b^	1.18 (1.13-1.24)	62.9^ b^	0.91 (0.84-0.99)
Some college/AD	64.8	(reference)	77.6^ b^	1.20 (1.17-1.23)	69.1^ b^	1.07 (1.04-1.10)	78.3^ b^	1.21 (1.17-1.25)	63.9	0.98 (0.94-1.03)
College graduate	55.3	(reference)	75.4^ b^	1.37 (1.34-1.41)	62.0^ b^	1.12 (1.08-1.16)	79.7^ b^	1.44 (1.41-1.47)	59.3^ b^	1.07 (1.04-1.10)

**Table 14:** Sex differences in the prevalence of hypertension within four levels of education, by race and ethnicity, among adults aged 65-79 years. %: age-standardized prevalence among adults with this level of education; aPR: adjusted prevalence ratio; CI: confidence interval; GED: high school diploma equivalent; AD: associate (community college) degree; College graduate: ≥ bachelor’s degree. ^a^ aPRs in this column compare the prevalence within this level of education between men and women after adjusting for age group. An aPR > 1.00 indicates a higher prevalence and an aPR < 1.00 indicates a lower prevalence among men than women if the 95% CI does not include 1.00. An aPR with a 95% CI that does not include 1.00 indicates that men were significantly different from women within the same level of education at p < 0.05 after adjusting for age group. ^b^ Meaningful difference in the prevalence at this level of education between men and women in this racial/ethnic group based on a ≥ 1 percentage point difference between subgroups and an aPR that is statistically significant at p < 0.05.

Level of education	White		Black		Latino		Filipino		Chinese	
	%	aPR^ a^ (95% CI)	%	aPR^ a^ (95% CI)	%	aPR^ a^ (95% CI)	%	aPR^ a^ (95% CI)	%	aPR^ a^ (95% CI)
Women										
Non-high school graduate	64.0	(reference)	85.6	(reference)	72.5	(reference)	84.4	(reference)	55.8	(reference)
High school graduate/GED	63.7	(reference)	85.0	(reference)	68.9	(reference)	80.3	(reference)	58.6	(reference)
Some college/AD	58.4	(reference)	81.7	(reference)	64.0	(reference)	80.5	(reference)	57.8	(reference)
College graduate	46.7	(reference)	75.5	(reference)	56.0	(reference)	77.7	(reference)	52.8	(reference)
Men										
Non-high school graduate	70.9^ b^	1.08 (1.04-1.12)	83.6	0.97 (0.92-1.02)	72.6	1.01 (0.97-1.06)	75.7^ b ^	0.89 (0.82-0.96)	62.8	1.11 (0.96-1.29)
High school graduate/GED	68.5^ b^	1.07 (1.05-1.09)	82.2	0.97 (0.93-1.00)	71.8^ b^	1.04 (1.00-1.09)	81.5	1.01 (0.95-1.07)	62.9	1.06 (0.96-1.19)
Some college/AD	64.8^ b^	1.11 (1.09-1.12)	77.6^ b^	0.95 (0.82-0.97)	69.1^ b^	1.09 (1.05-1.13)	78.3	0.97 (0.93-1.01)	63.9^ b^	1.11 (1.04-1.19)
College graduate	55.3^ b^	1.19 (1.17-1.20)	75.4	1.00 (0.97-1.03)	62.0^ b^	1.11 (1.06-1.17)	79.7	1.03 (1.00-1.05)	59.3^ b^	1.14 (1.09-1.20)

Association of educational attainment with the prevalence of coronary artery disease

The overall prevalence of CAD in the full sample was 3.2% among women and 7.7% among men. Across racial/ethnic groups, the overall prevalence ranged from 2.3% to 5.8% among women and 5.5% to 8.9% among men, with Chinese women and men having the lowest prevalence and Black women and Filipino men having the highest prevalence (Table 2). Compared to college graduates, the prevalence of CAD was significantly higher at the three lower levels of education among adults in the full sample and among White adults and Black women (Table 15), with differences seen between the lower and highest levels among Latino adults. There was minimal variation in prevalence across levels of education among Latino, Filipino, and Chinese adults. The largest differences in prevalence were seen among White and Black women non-high school graduates compared to their college graduate counterparts (6.4% vs. 2.2% and 9.5% vs. 4.3%, respectively). No step gradient relationships were seen, and non-step gradient relationships were only seen for the full sample and for White, Black, and Latino adults.

**Table 15:** Comparison of the prevalence of coronary artery disease at four levels of education between women and men aged 65-79 years. %: age-standardized prevalence among adults with this level of education; in the "Full sample" column, the age-standardization model prevalence additionally controlled for racial/ethnic group; aPR: adjusted prevalence ratio; CI: confidence interval; GED: high school diploma equivalent; AD: associate degree; College graduate: ≥ bachelor’s degree. ^a^ aPRs in this column compare prevalence at the lower three levels of education to the prevalence at the ≥ bachelor’s degree level. An aPR > 1.00 indicates a higher prevalence at the lower level of education compared to the prevalence at the college graduate level, while an aPR < 1.00 indicates a lower prevalence compared to that at the college graduate level. An aPR with a 95% CI that does not include 1.00 is statistically significant at p < 0.05. ^b^ The prevalence among adults at this level of education significantly (p < 0.05) differs from the prevalence among adults at the next higher level based on an aPR that adjusted for age group (65-69, 70-74, 75-79 years); for the "Full sample," the aPRs adjusted for age group and racial/ethnic group. ^c^ The prevalence among adults at this level of education significantly (p < 0.05) differs from the prevalence among college graduates after adjusting for age group; for the "Full sample," the aPR adjusted for age group and racial/ethnic group. ^d^ The prevalence among adults in this racial/ethnic group significantly (p < 0.05) differs from the prevalence among White adults with the same level of education after adjusting for age group. ^e^ The prevalence among Filipino adults significantly (p < 0.05) differs from the prevalence among Chinese adults with the same level of education after adjusting for age group. ^f^ The prevalence among women significantly (p < 0.05) differs from the prevalence among men at the same level of education and of the same racial/ethnic group; for the "Full sample," the aPR testing for sex differences adjusted for age group and racial/ethnic group.

Level of education	Full sample		White		Black		Latino		Filipino		Chinese	
	%	aPR^ a^ (95% CI)	%	aPR^ a^ (95% CI)	%	aPR^ a^ (95% CI)	%	aPR^ a^ (95% CI)	%	aPR^ a^ (95% CI)	%	aPR^ a^ (95% CI)
Women												
Non-high school graduate	5.7^ b^	2.26^ c^ (2.01-2.55)	6.4^ b^	2.84^ c^ (2.43-3.33)	9.5^ bd^	2.10^ c^ (1.54-2.86)	5.6	1.66^ c^ (1.20-2.29)	4.6	1.07 (0.69-1.65)	3.1^ d^	1.67 (0.79-3.52)
High school graduate/GED	4.2	1.74^ c^ (1.53-1.90)	4.3	1.90^ c^ (1.71-2.10)	6.6^ d^	1.49^ c^ (1.15-1.94)	4.3	1.30 (0.95-1.78)	5.1	1.13 (0.75-1.70)	3.5	1.91^ c^ (1.09-3.35)
Some college/AD	3.5^ b^	1.48^ c^ (1.37-1.59)	3.5^ b^	1.57^ c^ (1.43-1.72)	6.0^ bd^	1.35^ c^ (1.10-1.67)	4.0	1.20 (0.88-1.64)	4.9^ de^	1.19 (0.89-1.59)	2.4	1.34 (0.82-2.19)
College graduate	2.3	(reference)	2.2	(reference)	4.3^ d^	(reference)	3.4^ d^	(reference)	4.2^ de^	(reference)	2.0	(reference)
Men												
Non-high school graduate	10.6^ bf^	1.52^ c^ (1.38-1.68)	11.5^ bf^	1.73^ c^ (1.53-1.96)	11.1	1.42^ c^ (1.04-1.94)	9.6^ f^	1.32^ c^ (1.05-1.67)	10.0^ f^	0.95 (0.64-1.41)	5.0^ d^	0.83 (0.44-1.55)
High school graduate/GED	9.2^ f^	1.33^ c^ (1.24-1.42)	9.4^ f^	1.39^ c^ (1.29-1.51)	7.9	1.08 (0.84-1.39)	9.7^ f^	1.34^ c^ (1.08-1.67)	10.4^ ef^	1.01 (0.76-1.33)	4.8^ d^	0.81 (0.50-1.32)
Some college/AD	8.5^ bf^	1.24^ c^ (1.17-1.30)	8.7^ bf^	1.29^ c^ (1.22-1.37)	8.0^ f^	1.08 (0.88-1.31)	8.2^ f^	1.14 (0.93-1.40)	8.6^ f^	0.84 (0.67-1.04)	8.2^ bf^	1.31^ c^ (1.01-1.70)
College graduate	6.9^ f^	(reference)	6.7^ f^	(reference)	7.4^ f^	(reference)	7.2^ f^	(reference)	10.3^ def^	(reference)	6.0^ f^	(reference)

The prevalence of CAD was higher among Black than White women at all four levels of education and higher among Filipino than White adults at the college graduate level (Tables 15, 16). Compared to Chinese adults, Filipino women had a higher prevalence of CAD at ≥ some college levels and men at the high school graduate and college graduate levels. CAD prevalence was higher among men than women across all levels of education among White, Latino, and Filipino adults and was higher among men than women among Black and Chinese adults at the some college and college graduate levels (Tables 15, 17).

**Table 16:** Comparison of the prevalence of coronary artery disease among racial/ethnic groups within the same level of education between women and men aged 65-79 years. %: age-standardized prevalence among adults with this level of education; aPR: adjusted prevalence ratio; CI: confidence interval; GED: high school diploma equivalent; AD: associate (community college) degree; College graduate: ≥ bachelor’s degree. ^a^ aPRs in this column compare the prevalence within the level of education between adults in this racial/ethnic group and the White group after adjusting for the age group. An aPR > 1.00 indicates a significantly higher prevalence and an aPR < 1.00 indicates a significantly lower prevalence in this racial/ethnic group compared to White adults if the 95% CI does not include 1.00. An aPR with a 95% CI that does not include 1.00 indicates that the between-group difference in the prevalence was statistically significant at p < 0.05 after adjusting for age group. ^b^ Meaningful difference in the prevalence at this level of education between adults in this racial/ethnic group compared to White adults based on a ≥ 1 percentage point difference between subgroups and an aPR that is statistically significant at p < 0.05.

Level of education	White		Black		Latino		Filipino		Chinese	
	%	aPR^ a ^(95% CI)	%	aPR^ a^ (95% CI)	%	aPR^ a^ (95% CI)	%	aPR^ a^ (95% CI)	%	aPR^ a^ (95% CI)
Women										
Non-high school graduate	6.4	(reference)	9.5^ b^	1.41 (1.05-1.88)	5.6	0.88 (0.69-1.13)	4.6	0.68 (0.44-1.03)	3.1^ b^	0.48 (0.24-0.96)
High school graduate/GED	4.3	(reference)	6.6^ b^	1.53 (1.24-1.89)	4.3	1.03 (0.84-1.27)	5.1	1.09 (0.75-1.58)	3.5	0.84 (0.53-1.33)
Some college/AD	3.5	(reference)	6.0^ b^	1.70 (1.49-1.95)	4.0	1.16 (0.95-1.41)	4.9^ b^	1.41 (1.10-1.81)	2.4	0.70 (0.48-1.03)
College graduate	2.2	(reference)	4.3^ b^	1.99 (1.65-2.39)	3.4^ b^	1.52 (1.18-1.96)	4.2^ b^	1.87 (1.57-2.22)	2.0	0.82 (0.60-1.12)
Men										
Non-high school graduate	11.5	(reference)	11.1	0.90 (0.67-1.21)	9.6	0.82 (0.67-1.01)	10.0	0.82 (0.55-1.22)	5.0^ b^	0.42 (0.23-0.78)
High school graduate/GED	9.4	(reference)	7.9	0.85 (0.69-1.06)	9.7	1.03 (0.88-1.22)	10.4	1.09 (0.85-1.42)	4.8^ b^	0.52 (0.32-0.83)
Some college/AD	8.7	(reference)	8.0	0.92 (0.81-1.06)	8.2	0.94 (0.82-1.09)	8.6	1.00 (0.83-1.21)	8.2	0.90 (0.72-1.13)
College graduate	6.7	(reference)	7.4	1.12 (0.96-1.30)	7.2	1.07 (0.91-1.26)	10.3^ b^	1.52 (1.35-1.73)	6.0	0.91 (0.78-1.06)

**Table 17:** Sex differences in the prevalence of coronary artery disease within four levels of education, by race and ethnicity, among adults aged 65-79 years. %: age-standardized prevalence among adults with this level of education; aPR: adjusted prevalence ratio; CI: confidence interval; GED: high school diploma equivalent; AD: associate (community college) degree; College graduate: ≥ bachelor’s degree. ^a^ aPRs in this column compare the prevalence within this level of education between men and women after adjusting for age group. An aPR > 1.00 indicates a higher prevalence and an aPR < 1.00 indicates a lower prevalence among men than women if the 95% CI does not include 1.00. An aPR with a 95% CI that does not include 1.00 indicates that men were significantly different from women within the same level of education at p < 0.05 after adjusting for age group. ^b^ Meaningful difference in the prevalence at this level of education between men and women in this racial/ethnic group based on a ≥ 1 percentage point difference between subgroups and an aPR that is statistically significant at p < 0.05.

Level of education	White		Black		Latino		Filipino		Chinese	
	%	aPR^ a^ (95% CI)	%	aPR^ a^ (95% CI)	%	aPR^ a^ (95% CI)	%	aPR^ a^ (95% CI)	%	aPR^ a^ (95% CI)
Women										
Non-high school graduate	6.4	(reference)	9.5	(reference)	5.6	(reference)	4.6	(reference)	3.1	(reference)
High school graduate/GED	4.3	(reference)	6.6	(reference)	4.3	(reference)	5.1	(reference)	3.5	(reference)
Some college/AD	3.5	(reference)	6.0	(reference)	4.0	(reference)	4.9	(reference)	2.4	(reference)
College graduate	2.2	(reference)	4.3	(reference)	3.4	(reference)	4.2	(reference)	2.0	(reference)
Men										
Non-high school graduate	11.5^ b^	1.80 (1.50-2.16)	11.1	1.15 (0.80-1.66)	9.6^ b^	1.68 (1.29-2.19)	10.0^ b^	2.08 (1.21-3.57)	5.0	1.60 (0.65-3.94)
High school graduate/GED	9.4^ b^	2.18 (1.97-2.42)	7.9	1.23 (0.92-1.63)	9.7^ b^	2.19 (1.71-2.79)	10.4^ b^	2.15 (1.37-3.36)	4.8	1.33 (0.69-2.54)
Some college/AD	8.7^ b^	2.47 (2.29-2.66)	8.0^ b^	1.35 (1.13-1.61)	8.2^ b^	2.02 (1.61-2.55)	8.6^ b^	1.75 (1.30-2.37)	8.2^ b^	3.21 (2.07-4.98)
College graduate	6.7^ b^	3.00 (2.77-3.24)	7.4	1.70 (1.35-2.13)	7.2^ b^	2.11 (1.58-2.82)	10.3^ b^	2.48 (2.03-3.03)	6.0^ b^	3.29 (2.35-4.61)

Association of educational attainment with the prevalence of chronic obstructive pulmonary disease

The overall prevalence of COPD in the full sample was 8.3% among women and 9.1% among men. Across racial/ethnic groups, the overall prevalence ranged from 2.4% to 9.6% among women and 4.1% to 10.9% among men, with Chinese adults having the lowest prevalence and Black adults having the highest prevalence (Table 2). The prevalence of COPD was significantly higher at the three lower levels of education than among college graduates in the full sample and among White, Black, and Latino adults and Filipino men (Table 18). Differences were also seen between Chinese and White adults at the high school graduate level and men with some college compared to college graduates. Compared to college graduates, the prevalence of COPD was 13% to three-fold higher among women and 46% to three-fold higher among men who were non-high school graduates, 24% to > two-fold higher among women and 58% to two-fold higher among men who were high school graduates, and 11% to nearly two-fold higher among women and 26% to 90% higher among men with some college. Step gradient relationships were seen for adults in the full sample and for White adults, and non-step gradient relationships were seen for Black, Latino, and Filipino adults.

**Table 18:** Comparison of the prevalence of chronic obstructive pulmonary disease (COPD) at four levels of education between women and men aged 65-79 years. %: age-standardized prevalence among adults with this level of education; in the "Full sample" column, the age-standardization model prevalence additionally controlled for racial/ethnic group; aPR: adjusted prevalence ratio; CI: confidence interval; GED: high school diploma equivalent; AD: associate degree; College graduate: ≥ bachelor’s degree. ^a^ aPRs in this column compare the prevalence at the lower three levels of education to the prevalence at the ≥ bachelor’s degree level. An aPR > 1.00 indicates a higher prevalence at the lower level of education compared to the prevalence at the college graduate level, while an aPR < 1.00 indicates a lower prevalence compared to that at the college graduate level. An aPR with a 95% CI that does not include 1.00 is statistically significant at p < 0.05. ^b^ The prevalence among adults at this level of education significantly (p < 0.05) differs from the prevalence among adults at the next higher level based on an aPR that adjusted for age group (65-69, 70-74, 75-79 years); for the "Full sample," the aPRs adjusted for age group and racial/ethnic group. ^c^ The prevalence among adults at this level of education significantly (p < 0.05) differs from the prevalence among college graduates level after adjusting for age group; for the "Full sample," the aPR adjusted for age group and racial/ethnic group. ^d^ The prevalence among adults in this racial/ethnic group significantly (p < 0.05) differs from the prevalence among White adults with the same level of education after adjusting for age group. ^e^ The prevalence among Filipino adults significantly (p < 0.05) differs from the prevalence among Chinese adults with the same level of education after adjusting for age group. ^f^ The prevalence among women significantly (p < 0.05) differs from the prevalence among men at the same level of education and of the same racial/ethnic group; for the "Full sample," the aPR testing for sex differences adjusted for age group and racial/ethnic group.

Level of education	Full sample		White		Black		Latino		Filipino		Chinese	
	%	aPR^ a ^(95% CI)	%	aPR^ a ^(95% CI)	%	aPR^ a^ (95% CI)	%	aPR^ a^ (95% CI)	%	aPR^ a^ (95% CI)	%	aPR^ a^ (95% CI)
Women												
Non-high school graduate	15.4^ b^	2.97^ c^ (2.75-3.22)	18.2^ b^	3.22^ c^ (2.94-3.53)	17.7^ b^	2.83^ c^ (2.24-3.56)	7.7^ bd^	2.20^ c^ (1.62-3.00)	5.8^ de^	1.61^ c^ (1.10-2.38)	2.2^ d^	1.13 (0.49-2.60)
High school graduate/GED	12.0^ b^	2.19^ c^ (2.07-2.32)	12.5^ b^	2.30^ c^ (2.16-2.45)	11.4	1.87^ c^ (1.52-2.29)	5.4^ d^	1.58^ c^ (1.17-2.14)	4.6^ d^	1.24 (0.82-1.89)	4.0^ d^	1.93^ c^ (1.14-3.26)
Some college/AD	9.8^ b^	1.80^ c^ (1.71-1.89)	9.9^ b^	1.86^ c^ (1.76-1.93)	10.1^ b^	1.65^ c^ (1.39-1.96)	4.8^ bd^	1.41^ c^ (1.05-1.90)	4.0^ de^	1.11 (0.81-1.54)	2.4^ d^	1.21 (0.75-1.94)
College graduate	5.8	(reference)	5.4	(reference)	6.2	(reference)	3.5^ d^	(reference)	3.5^ de^	(reference)	2.1^ d^	(reference)
Men												
Non-high school graduate	16.5^ bf^	2.79^ c^ (2.57-3.04)	18.4^ b^	3.03^ c^ (2.74-3.35)	20.0	2.54^ c^ (1.98-3.24)	9.6^ df^	2.25^ c^ (1.71-2.97)	12.7^ def^	1.72^ c^ (1.21-2.44)	4.4^ d^	1.46 (0.74-2.88)
High school graduate/GED	14.7^ bf^	2.45^ c^ (2.31-2.60)	15.4^ bf^	2.61^ c^ (2.44-2.79)	15.5^ bf^	2.07^ c^ (1.68-2.54)	7.5^ df^	1.85^ c^ (1.41-2.44)	12.1^ def^	1.58^ c^ (1.20-2.07)	6.8^ d^	2.22^ c^ (1.43-3.46)
Some college/AD	10.8^ bf^	1.80^ c^ (1.71-1.90)	11.1^ bf^	1.90^ c^ (1.79-2.01)	10.7^ b^	1.43^ c^ (1.18-1.72)	6.1^ bdf^	1.51^ c^ (1.16-1.96)	9.3^ bef^	1.26^ c^ (1.01-1.57)	6.1^ bdf^	1.87^ c^ (1.34-2.61)
College graduate	6.2	(reference)	5.8	(reference)	7.5^ df^	(reference)	4.1^ d^	(reference)	7.4^ def^	(reference)	3.1^ df^	(reference)

The prevalence of COPD was similar for White and Black women at all levels of education and for White and Black men at the three lower levels of education, but higher among Black than White men at the college graduate level. Compared to White adults, COPD prevalence was lower among Latino and Chinese adults and Filipina women at all levels of education. The prevalence was lower among Filipino men than in White men at the two lower levels of education but higher at the college graduate level (Tables 18, 19). Compared to Chinese adults, Filipino men at all levels of education and Filipino women at non-high school graduate and some college levels were more likely to have COPD. The prevalence of COPD was higher among men than women among Filipino adults at all levels of education, Latino adults at all but the college graduate level, and White and Black adults at the high school graduate level (Tables 18, 20).

**Table 19:** Comparison of the prevalence of chronic obstructive pulmonary disease (COPD) among racial/ethnic groups within the same level of education between women and men aged 65-79 years. %: age-standardized prevalence among adults with this level of education; aPR: adjusted prevalence ratio; CI: confidence interval; GED: high school diploma equivalent; AD: associate (community college) degree; College graduate: ≥ bachelor’s degree. ^a^ aPRs in this column compare the prevalence within this level of education between adults in this racial/ethnic group and the White group after adjusting for the age group. An aPR > 1.00 indicates a significantly higher prevalence and an aPR < 1.00 indicates a significantly lower prevalence in this racial/ethnic group compared to White adults if the 95% CI does not include 1.00. An aPR with a 95% CI that does not include 1.00 indicates that the between-group difference in the prevalence was statistically significant at p < 0.05 after adjusting for age group. ^b^ Meaningful difference in the prevalence at this level of education between adults in this racial/ethnic group compared to White adults based on a ≥ 1 percentage point difference between subgroups and an aPR that is statistically significant at p < 0.05.

Level of education	White		Black		Latino		Filipino		Chinese	
	%	aPR^ a^ (95% CI)	%	aPR^ a^ (95% CI)	%	aPR^ a^ (95% CI)	%	aPR^ a^ (95% CI)	%	aPR^ a^ (95% CI)
Women										
Non-high school graduate	18.2	(reference)	17.7	0.95 (0.78-1.16)	7.7^ b^	0.43 (0.35-0.52)	5.8^ b^	0.34 (0.24-0.48)	2.2^ b^	0.13 (0.06-0.29)
High school graduate/GED	12.5	(reference)	11.4	0.91 (0.78-1.06)	5.4^ b^	0.43 (0.36-0.52)	4.6^ b^	0.37 (0.25-0.53)	4.0^ b^	0.32 (0.21-0.49)
Some college/AD	9.9	(reference)	10.1	1.03 (0.94-1.14)	4.8^ b^	0.48 (0.41-0.57)	4.0^ b^	0.40 (0.30-0.52)	2.4^ b^	0.25 (0.17-0.36)
College graduate	5.4	(reference)	6.2	1.18 (1.01-1.37)	3.5^ b^	0.65 (0.51-0.83)	3.5^ b^	0.66 (0.55-0.79)	2.1^ b^	0.39 (0.29-0.52)
Men										
Non-high school graduate	18.4	(reference)	20.0	1.06 (0.86-1.32)	9.6^ b^	0.52 (0.43-0.64)	12.7^ b^	0.71 (0.50-0.98)	4.4^ b^	0.24 (0.13-0.46)
High school graduate/GED	15.4	(reference)	15.5	1.00 (0.86-1.17)	7.5^ b^	0.50 (0.42-0.60)	12.1^ b^	0.77 (0.61-0.97)	6.8^ b^	0.45 (0.30-0.66)
Some college/AD	11.1	(reference)	10.7	0.97 (0.86-1.09)	6.1^ b^	0.56 (0.47-0.66)	9.3	0.85 (0.71-1.01)	6.1^ b^	0.52 (0.40-0.67)
College graduate	5.8	(reference)	7.5^ b^	1.29 (1.11-1.51)	4.1^ b^	0.71 (0.58-0.88)	7.4^ b^	1.27 (1.10-1.47)	3.1^ b^	0.53 (0.42-0.65)

**Table 20:** Sex differences in the prevalence of chronic obstructive pulmonary disease (COPD) within four levels of education, by race and ethnicity, among adults aged 65-79 years. %: age-standardized prevalence among adults with this level of education; aPR: adjusted prevalence ratio; CI: confidence interval; GED: high school diploma equivalent; AD: associate (community college) degree; College graduate: ≥ bachelor’s degree. ^a^ aPRs in this column compare the prevalence within this level of education between men and women after adjusting for age group. An aPR > 1.00 indicates a higher prevalence and an aPR < 1.00 indicates a lower prevalence among men than women if the 95% CI does not include 1.00. An aPR with a 95% CI that does not include 1.00 indicates that men were significantly different from women within the same level of education at p < 0.05 after adjusting for age group. ^b^ Meaningful difference in the prevalence at this level of education between men and women in this racial/ethnic group based on a ≥ 1 percentage point difference between subgroups and an aPR that is statistically significant at p < 0.05.

Level of education	White		Black		Latino		Filipino		Chinese	
	%	aPR^ a^ (95% CI)	%	aPR^ a^ (95% CI)	%	aPR^ a^ (95% CI)	%	aPR^ a^ (95% CI)	%	aPR^ a^ (95% CI)
Women										
Non-high school graduate	18.2	(reference)	17.7	(reference)	7.7	(reference)	5.8	(reference)	2.2	(reference)
High school graduate/GED	12.5	(reference)	11.4	(reference)	5.4	(reference)	4.6	(reference)	4.0	(reference)
Some college/AD	9.9	(reference)	10.1	(reference)	4.8	(reference)	4.0	(reference)	2.4	(reference)
College graduate	5.4	(reference)	6.2	(reference)	3.5	(reference)	3.5	(reference)	2.1	(reference)
Men										
Non-high school graduate	18.4	1.01 (0.89-1.14)	20.0	1.13 (0.87-1.47)	9.6	1.23 (0.96-1.58)	12.7^ b^	2.09 (1.31-3.33)	4.4	1.95 (0.72-5.29)
High school graduate/GED	15.4^ b^	1.23 (1.15-1.32)	15.5^ b^	1.37 (1.12-1.68)	7.5^ b^	1.41 (1.11-1.80)	12.1^ b^	1.64 (1.70-4.12)	6.8	1.72 (0.96-3.06)
Some college/AD	11.1^ b^	1.11 (1.06-1.17)	10.7	1.05 (0.91-1.21)	6.1^ b^	1.28 (1.02-1.62)	9.3^ b^	2.39 (1.73-3.29)	6.1^ b^	2.28 (1.45-3.61)
College graduate	5.8	1.09 (1.02-1.15)	7.5	1.20 (0.98-1.48)	4.1	1.19 (0.87-1.65)	7.4^ b^	2.10 (1.67-2.62)	3.1^ b^	1.47 (1.03-2.10)

Impact of adjusting for educational attainment when comparing the prevalence of health outcomes among racial/ethnic groups and women and men

We examined whether using educational attainment as a risk adjustment covariate attenuated differences between White adults and adults in the other racial/ethnic groups for the six health outcomes by comparing aPRs from sex-specific models that only controlled for age group with aPRs from models that controlled for age group and educational attainment. Differences between aPR pairs were defined as meaningfully significant when there was a difference of ≥ 10% between the two aPRs (e.g., aPR = 1.05 vs. 1.15) with non-overlapping 95% CIs or when an aPR that adjusted only for age became non-statistically significant after additionally adjusting for education. Adjusting for education had the most robust impact on modifying gaps in prevalence between Latino and White adults, though changes were not always in the same direction. Specifically, adjusting for education increased differences between Latino and White adults in the prevalence of current smoking and COPD and decreased differences in the prevalence of diabetes (Table 21). Adjusting for education also resulted in a few differences for Black and White adults, decreasing differences in the prevalence of current smoking and diabetes among men and COPD among women and men. Adjusting for education did not result in any meaningful changes in aPRs comparing White adults to Filipino and Chinese adults. Adjusting for education also had no meaningful impact on comparisons of prevalence between Filipino and Chinese adults and between women and men for any of the health outcomes (data not shown).

**Table 21:** Impact of adjusting for level of education and age group versus age group alone on comparisons of prevalence of six health outcomes among Black, Latino, Chinese and Filipino adults versus White adults aged 65-79 years. aPR: adjusted prevalence ratio (PR) comparing the prevalence of health outcome among adults in this racial/ethnic group to the prevalence in the White group after adjusting for covariates; CI: confidence interval; Age group: ages 65-69 years, 70-74 years, and 75-79 years (reflecting the age groups used to produce age-standardized prevalence estimates); Education: non-high school graduate/high school graduate/some college or associate degree/≥ bachelor’s degree. An aPR > 1.00 indicates a higher prevalence and an aPR < 1.00 indicates a lower prevalence in this racial/ethnic group compared to the White group if the 95% CI does not include 1.00. An aPR pair with a ≥ 10% difference between the aPR that only adjusted for age and the aPR that additionally adjusted for education (e.g., 1.10 vs. 1.25) was considered meaningfully different. Note also that in some instances, adjusting for education resulted in the difference between adults in this racial/ethnic group and White adults becoming non-statistically significant.

Modeled health outcome	Black	Latino	Filipino	Chinese
	aPR (95% CI)	aPR (95% CI)	aPR (95% CI)	aPR (95% CI)
Women				
Ever smoking				
Age group only	1.04 (1.01-1.06)	0.76 (0.73-0.78)	0.32 (0.30-0.34)	0.28 (0.26-0.31)
Age group + education	1.00 (0.98-1.03)	0.71 (0.68-0.73)	0.33 (0.31-.035)	0.29 (0.27-0.31)
Current smoking				
Age group only	1.58 (1.47-1.70)	0.82 (0.74-0.92)	0.46 (0.39-0.55)	0.30 (0.24-0.38)
Age group + education	1.43 (1.33-1.54)	0.65 (0.58-0.73)	0.49 (0.41-0.58)	0.31 (0.25-0.40)
Diabetes				
Age group only	2.24 (2.17-2.31)	2.11 (2.04-2.19)	2.80 (2.71-2.90)	1.37 (1.29-1.45)
Age group + education	2.12 (2.05-2.20)	1.87 (1.80-1.94)	2.92 (2.82-3.02)	1.41 (1.33-1.50)
Hypertension				
Age group only	1.50 (1.48-1.51)	1.20 (1.18-1.22)	1.46 (1.44-1.49)	1.01 (0.98-1.04)
Age group + education	1.46 (1.44-1.47)	1.13 (1.11-1.15)	1.50 (1.48-1.53)	1.03 (1.00-1.06)
Coronary artery disease				
Age group only	1.83 (1.67-2.01)	1.36 (1.22-1.52)	1.39 (1.23-1.58)	0.73 (0.59-0.89)
Age group + education	1.72 (1.57-1.88)	1.15 (1.02-1.28)	1.44 (1.27-1.63)	0.75 (0.61-0.92)
Chronic obstructive pulmonary disease				
Age group only	1.13 (1.06-1.21)	0.61 (0.55-0.67)	0.46 (0.41-0.53)	0.28 (0.23-0.34)
Age group + education	1.03 (0.97-1.10)	0.48 (0.44-0.53)	0.48 (0.43-0.55)	0.30 (0.24-0.36)
Men				
Ever smoking				
Age group only	1.12 (1.09-1.14)	1.01 (0.99-1.03)	1.12 (1.09-1.15)	0.57 (0.54-0.60)
Age group + education	1.04 (1.01-1.06)	0.91 (0.89-0.94)	1.11 (1.08-1.14)	0.59 (0.56-0.61)
Current smoking				
Age group only	1.72 (1.58-1.86)	1.01 (0.91-1.12)	1.07 (0.94-1.21)	0.57 (0.48-0.67)
Age group + education	1.46 (1.34-1.58)	0.78 (0.71-0.87)	1.03 (0.91-1.16)	0.60 (0.51-0.71)
Diabetes				
Age group only	1.73 (1.67-1.79)	1.76 (1.71-1.82)	2.29 (2.22-2.37)	1.27 (1.21-1.34)
Age group + education	1.61 (1.56-1.67)	1.59 (1.54-1.65)	2.27 (2.20-2.35)	1.31 (1.24-1.37)
Hypertension				
Age group only	1.30 (1.28-1.32)	1.14 (1.12-1.15)	1.32 (1.30-1.34)	1.01 (0.98-1.03)
Age group + education	1.26 (1.24-1.28)	1.09 (1.07-1.10)	1.31 (1.29-1.34)	1.02 (1.00-1.05)
Coronary artery disease				
Age group only	1.03 (0.95-1.13)	1.09 (1.01-1.18)	1.26 (1.15-1.38)	0.81 (0.72-0.92)
Age group + education	0.98 (0.89-1.07)	0.99 (0.91-1.08)	1.25 (1.14-1.37)	0.83 (0.73-0.93)
Chronic obstructive pulmonary disease				
Age group only	1.23 (1.14-1.33)	0.72 (0.66-0.79)	0.99 (0.90-1.09)	0.45 (0.39-0.53)
Age group + education	1.06 (0.99-1.15)	0.56 (0.51-0.62)	0.97 (0.88-1.07)	0.48 (0.41-0.56)

## Discussion

This study is among the first to investigate the education-health relationship in a contemporary community-dwelling older US adult population and how this relationship varies across racial/ethnic groups and health outcomes. Most previous studies of US adults have used very geographically and age-diverse study populations that sometimes even excluded older adults, and when examining for racial/ethnic differences, have restricted analyses to differences between White, Black, and sometimes Hispanic/Latino adults. Our research adds to existing knowledge by providing information about the association of educational attainment with multiple health outcomes overall and within racial/ethnic groups in an older adult population and by expanding the racial/ethnic groups studied to include US Filipino and Chinese adults, the two largest Asian ethnic groups in the US that are usually aggregated under the umbrella of Asian race. Additionally, most previous studies have only modeled how risk or odds of having a health behavior or health condition varies by level of education overall and within selected racial/ethnic groups and used interaction terms to test for racial/ethnic differences. In our study, in addition to using adjusted prevalence ratios to compare prevalence between levels of education and by racial/ethnic group within the level of education, we also produced point estimates of the prevalence of health outcomes for women and men in our five racial/ethnic groups at non-high school graduate, high school graduate, some college, and college graduate levels that facilitate understanding of the absolute magnitude of differences between subgroups in these comparisons.

Our study showed that the association of educational attainment with smoking and health status in our older adult study population varied across health outcomes and that the gradient pattern of association was more consistent for White and Black than Latino, Filipino, and Chinese older adults (Table 22). Specifically, we found gradient relationships of level of education with all six health outcomes (ever smoking, current smoking, diabetes, hypertension, CAD, and COPD) for White and Black adults, with prevalence of the smoking and chronic health conditions decreasing as level of education increased. However, that gradient relationship did not always have a step pattern (i.e., consistent increase or decrease at each adjacent level of education). Among Latino adults, we saw a gradient relationship for current smoking, diabetes, hypertension (women only), and COPD. However, the prevalence of ever smoking only significantly differed between college graduates and the three lower levels, and for CAD, between college graduates and the two lowest levels.

**Table 22:** Summary of the patterns of association of educational attainment with the prevalence of smoking and chronic conditions among White, Black, Latino, Filipino, and Chinese adults aged 65-79 years. CAD: coronary artery disease; COPD: chronic obstructive pulmonary disease. ^a^ The prevalence at most or all levels of education below ≥ bachelor’s degree level is significantly higher compared to college graduate level. ^b^ The prevalence consistently decreases or increases compared to the next higher level of education for all levels of education (gradient). ^c^ The prevalence decreases or increases as educational attainment increases but does not consistently differ between each next higher level of education (upward or downward trend). ^d^ The prevalence only significantly differs between the lowest levels of education (non-high school graduate and/or high school graduate levels) and ≥ bachelor’s degree level. ^e^ The prevalence is similar across all levels of education or there is no consistent pattern of decreasing or increasing prevalence as educational attainment increases.

Racial/ethnic group	Ever smoker	Current smoker	Diabetes	Hypertension	CAD	COPD
Women						
Full sample	a, c	a, b	a, b	a, c	a, c	a, b
White	a, b	a, b	a, b	a, c	a, b	a, b
Black	a, c	a, c	a, c	a, c	a, c	a, c
Latina	a	a, c	a, b	a, b	d	a, c
Filipina	a	a	a, c	d	e	d
Chinese	e	d	a, c	e	e	e
Men						
Full sample	a, c	a, b	a, b	a, c	a, c	a, b
White	a, c	a, b	a, b	a, c	a, c	a, b
Black	a, c	a, c	a, c	a, c	d	a, c
Latino	a	a, c	a, b	a	d	a, c
Filipino	a	a	e	e	e	a, c
Chinese	a	a, c	d	e	e	e

Compared to patterns observed for the other racial/ethnic groups, Filipino and Chinese adults exhibited less consistent patterns of association of prevalence with levels of education, smaller absolute differences in prevalence between college graduates and non-high school graduates, and more sex differences in the prevalence of health outcomes. Among Filipina women, we only observed a gradient relationship for diabetes, but college graduates were less likely than those at all lower levels to be current or ever smokers and were less likely to have COPD than those at the two lowest levels. Among Filipino men, we saw a gradient relationship for COPD, and college graduates were less likely to be current and ever smokers than adults at the three lower levels of education, but we observed no consistent differences across levels of education for the prevalence of diabetes, hypertension, or CAD. Among Chinese women, we saw a gradient relationship for diabetes and found a lower prevalence of current smoking at the college graduate versus the two lowest levels of education. Among Chinese men, we observed a gradient relationship for current smoking, a higher prevalence of ever smoking at all levels compared to college graduates, and a higher prevalence of diabetes at the two lowest levels compared to college graduates. We found no consistent pattern of association of educational attainment with the prevalence of hypertension, CAD, or COPD among Chinese women and men.

Consistent with previous research [7,11-13], we found significant racial/ethnic differences in the prevalence of health outcomes among adults with the same level of education. For example, at all four levels of education, Black adults were more likely than White adults to be current smokers and to have diabetes and hypertension, and Black women were more likely than White women to have CAD. While the prevalence of ever smoking was the same for Black and White women and similar or only slightly higher for Black compared to White men at all four levels of education, Black adults at or above the high school graduate level were less likely than their White and Latino counterparts to have quit smoking by 2016. These findings of a lesser protective effect of higher educational attainment for Black adults compared to White adults for multiple health outcomes is in line with the MDR theory posited by Assari and others [15-17]. Other notable racial/ethnic differences found within most levels of education included a lower prevalence of current and ever smoking and concomitant lower prevalence of COPD among Latina and Filipina women and Chinese adults compared to their White counterparts and a higher prevalence of diabetes and hypertension among Latino and Filipino adults compared to White adults.

In this older adult study population, the prevalence of chronic health conditions among men and women in the same racial/ethnic group seldom significantly differed within the level of education. The largest sex differences within the level of education observed were for current and ever smoking among Filipino and Chinese adults and CAD among White, Latino, Filipino, and Chinese adults, with prevalence being higher among men. Additionally, because the distribution of education was fairly similar among women and men within the same racial/ethnic group, adjusting for age group and education level versus age group alone when comparing the prevalence of a health outcome among women versus men yielded similar prevalence ratios. However, this would likely be different in more age-diverse study populations, populations with greater sex differences in educational attainment, and populations with wider gaps between men and women in the overall prevalence of the health outcomes being investigated.

Strengths and limitations

Our study had several strengths. First, our very large racial/ethnically diverse study sample enabled us to compare age-standardized point prevalence of health outcomes across four levels of education separately for women and men within the five racial/ethnic groups and to compare prevalence among racial/ethnic groups within the same level of education. Second, our study furnishes data on the health of under-studied Filipino and Chinese older adults. Third, our health outcomes were derived from electronic health record diagnosis data rather than self-reported data. Fourth, we studied the association of educational attainment with multiple health outcomes. Finally, in contrast to most previous research on the education-health relationship that has utilized data for predominantly younger and middle-aged adult populations, our study focused on older adults.

We acknowledge, however, that our study has several limitations that can affect the generalizability of the results. First, because this was a cross-sectional study, we cannot make causal inferences between educational attainment earlier in life with health outcomes at older ages. Second, we lacked information about the quality of formal primary and secondary education received by adults in the sample and the country in which that education was received. Third, we lacked information about other social, behavioral, and environmental determinants of health earlier in life, including diet and other health-related behaviors, health literacy, social and physical environmental exposures and stressors, and access to health care. Fourth, we lacked information about the country of birth and the number of adult years spent in the US. Fifth, because of the paucity of educational attainment data for adults whose preferred language was not English, our study cohort excluded adults whose preferred spoken and written language was not English. While under 1% of White and Black adults and 5.6% of Filipino adults were excluded from the starting DECKA2016 [29] cohort due to language preference, 28% of Latino and 38% of Chinese adults were excluded. English spoken language preference is often used as a proxy indicator of acculturation in EHR-based studies, so the restriction to English preference may have eliminated older adults who were more recent immigrants or less acculturated. However, because English has been the official second language in the Philippines for many years, for Filipino adults, an English language preference in the EHR does not indicate greater acculturation to the same extent that it might for Latino and Chinese adults. Sixth, a larger percentage of Latino and Filipino adults than White, Black, and Chinese adults were missing education data and thus were excluded from our analyses. Seventh, our study population was fairly well educated, yielding smaller subgroups of adults at the non-high school graduate and high school graduate levels, especially in the Filipino and Chinese groups. This resulted in low statistical power to detect differences between these two lower levels and the higher levels of education and differences between adults in the White and the other racial/ethnic groups within these two levels of education. Finally, while we consider the relative homogeneity of the study population with regard to geography and healthcare access as a strength, this may limit the generalizability of our results to uninsured adults and adults residing in other geographic regions of the US.

## Conclusions

In this older-aged Northern California health plan population with a preferred spoken and written language of English, the association of educational attainment with health outcomes varied across racial/ethnic groups, with a less consistent gradient pattern seen for Filipino and Chinese adults than White, Black, and Latino older adults. Further, while the existence of a gradient pattern of the education-health relationship varied across health outcomes, the prevalence of the health outcome was almost always lower among college graduates compared to adults at the three lower levels of education and was often similar among high school graduates and those with some college. This suggests that when using education as a dichotomous covariate or risk adjustor in a relatively well-educated multi-ethnic older adult population, the cut-point should be college graduate versus lower levels of education rather than ≥ some college versus ≤ high school graduate.

Finally, while higher educational attainment was associated with a better health profile, controlling for educational attainment minimally attenuated differences in the overall prevalence of health outcomes when comparing White adults to adults in other racial/ethnic groups. This suggests that a four-level ordinal educational attainment variable may not work as well for risk adjustment in a US multi-ethnic older population that includes older adults who likely immigrated to the US as adults. Future research is needed to identify behavioral, cultural, and environmental life course factors beyond educational attainment that contribute to the higher prevalence of chronic health conditions among older adults of Black, Latino, and Asian race/ethnicity compared to White older adults so that culturally responsive screening protocols and interventions can be developed and implemented to achieve health equity.

## References

[1] Zimmerman E, Woolf SH (2014). Understanding the relationship between education and health. NAM Perspectives.

[2] Cutler DM, Lleras-Muney A (2006). Education and health: evaluating theories and evidence. NBER Working Paper Series, Working Paper 12352.

[3] Zajacova A, Lawrence EM (2018). The relationship between education and health: reducing disparities through a contextual approach. Annu Rev Public Health.

[4] Telfair J, Shelton TL (2012). Educational attainment as a social determinant of health. NC Med J.

[5] Egerter S, Braveman P, Sadegh-Nobari T, Grossman-Kahn R, Dekker M (2024). Education and Health. Exploring the social determinants of health: issue brief no. 5..

[6] Olshansky SJ, Antonucci T, Berkman L (2012). Differences in life expectancy due to race and educational differences are widening, and many may not catch up. Health Aff (Millwood).

[7] Johnson AE, Herbert BM, Stokes N, Brooks MM, Needham BL, Magnani JW (2022). Educational attainment, race, and ethnicity as predictors for ideal cardiovascular health: from the National Health and Nutrition Examination Survey. J Am Heart Assoc.

[8] Kaplan RM, Spittel ML, Zeno TL (2014). Educational attainment and life expectancy. Policy Insights Behav Brain Sci.

[9] Committee on the Recommended Social and Behavioral Domains and Measures for Electronic Health Records, Board on Population Health and Public Health Practice, Institute of Medicine (2015). Capturing Social and Behavioral Domains and Measures in Electronic Health Records: Phase 2. https://pubmed.ncbi.nlm.nih.gov/25590118/.

[10] National Academies of Sciences, Engineering Engineering, and Medicine (NASEM) (2016). Accounting for Social Risk Factors in Medicare Payment: Data.

[11] Dinwiddie GY, Zambrana RE, Doamekpor LA, Lopez L (2016). The impact of educational attainment on observed race/ethnic disparities in inflammatory risk in the 2001-2008 National Health and Nutrition Examination Survey. Int J Environ Res Public Health.

[12] Bell CN, Sacks TK, Thomas Tobin CS, Thorpe RJ Jr (2020). Racial non-equivalence of socioeconomic status and self-rated health among African Americans and Whites. SSM Popul Health.

[13] Braveman PA, Cubbin C, Egerter S, Williams DR, Pamuk E (2010). Socioeconomic disparities in health in the United States: what the patterns tell us. Am J Public Health.

[14] Kimbro RT, Bzostek S, Goldman N, Rodríguez G (2008). Race, ethnicity, and the education gradient in health. Health Aff (Millwood).

[15] Assari S (2018). Health disparities due to diminished return among Black Americans: public policy solutions. Soc Issues Policy Rev.

[16] Assari S, Cobb S, Saqib M, Bazargan M (2020). Diminished returns of educational attainment on heart disease among Black Americans. Open Cardiovasc Med J.

[17] Assari S, Cobb S, Najand B, Zare H, Sonnega A (2024). Race, educational attainment, and sustained high body mass index over 24 years of follow-up in middle-aged and older adults. J Racial Ethn Health Disparities.

[18] Choi K, Jones JT, Ruybal AL, McNeel TS, Duarte DA, Webb Hooper M (2023). Trends in education-related smoking disparities among U.S. Black or African American and White adults: intersections of race, sex, and region. Nicotine Tob Res.

[19] Agaku IT, Odani S, Okuyemi KS, Armour B (2020). Disparities in current cigarette smoking among US adults, 2002-2016. Tob Control.

[20] Nguyen-Grozavu FT, Pierce JP, Sakuma KK (2020). Widening disparities in cigarette smoking by race/ethnicity across education level in the United States. Prev Med.

[21] Choi JY, Choi D, Mehta NK, Ali MK, Patel SA (2024). Diabetes disparities in the United States: trends by educational attainment from 2001 to 2020. Am J Prev Med.

[22] Whitaker SM, Bowie JV, McCleary R, Gaskin DJ, LaVeist TA, Thorpe RJ Jr (2014). The association between educational attainment and diabetes among men in the United States. Am J Mens Health.

[23] Steele CJ, Schöttker B, Marshall AH (2017). Education achievement and type 2 diabetes-what mediates the relationship in older adults? Data from the ESTHER study: a population-based cohort study. BMJ Open.

[24] Zacher M (2023). Educational disparities in hypertension prevalence and blood pressure percentiles in the Health and Retirement Study. J Gerontol B Psychol Sci Soc Sci.

[25] Magnani JW, Ning H, Wilkins JT, Lloyd-Jones DM, Allen NB (2024). Educational attainment and lifetime risk of cardiovascular disease. JAMA Cardiol.

[26] Kubota Y, Heiss G, MacLehose RF, Roetker NS, Folsom AR (2017). Association of educational attainment with lifetime risk of cardiovascular disease: the atherosclerosis risk in communities study. JAMA Intern Med.

[27] Assari S, Chalian H, Bazargan M (2019). High education level protects European Americans but not African Americans against chronic obstructive pulmonary disease: National Health Interview Survey. Int J Biomed Eng Clin Sci.

[28] Gordon NP (2024). Similarity of adult Kaiser Permanente members to the adult population in Kaiser Permanente’s Northern California service area: comparisons based on the 2017/2018 cycle of the California Health Interview Survey. Kaiser Permanente Division of Research, Oakland, CA.

[29] Gordon NP, Lin TY, Rau J, Lo JC (2019). Aggregation of Asian-American subgroups masks meaningful differences in health and health risks among Asian ethnicities: an electronic health record based cohort study. BMC Public Health.

[30] Karter AJ, Schillinger D, Adams AS, Moffet HH, Liu J, Adler NE, Kanaya AM (2013). Elevated rates of diabetes in Pacific Islanders and Asian subgroups: the Diabetes Study of Northern California (DISTANCE). Diabetes Care.

